# Whole‐genome duplications followed by tandem duplications drive diversification of the protein modifier SUMO in Angiosperms

**DOI:** 10.1111/nph.13911

**Published:** 2016-03-02

**Authors:** Valentin Hammoudi, Georgios Vlachakis, M. Eric Schranz, Harrold A. van den Burg

**Affiliations:** ^1^ Molecular Plant Pathology Swammerdam Institute for Life Sciences University of Amsterdam Science Park 904 1089 XH Amsterdam the Netherlands; ^2^ Biosystematics Group Wageningen University Droevendaalsesteeg 1 6708 PB Wageningen the Netherlands

**Keywords:** evolution, immunity, neofunctionalization, palaeoploidy, paralogue, protein modification, SUMO, ubiquitin‐like modifier

## Abstract

- The ubiquitin‐like modifier (UBL) SUMO (Small Ubiquitin‐Like Modifier) regulates protein function. Structural rather than sequence homology typifies UBL families. However, individual UBL types, such as SUMO, show remarkable sequence conservation. Selection pressure also operates at the *SUMO* gene copy number, as increased SUMO levels activate immunity and alter flowering time in Arabidopsis.
- We show how, despite this selection pressure, the SUMO family has diversified into eight paralogues in Arabidopsis. Relationships between the paralogues were investigated using genome collinearity and gene tree analysis. We show that palaeopolyploidy followed by tandem duplications allowed expansion and then diversification of the *SUMO* genes.
- For example, Arabidopsis *SUMO5* evolved from the pan‐eudicot palaeohexaploidy event (gamma), which yielded three *SUMO* copies. Two gamma copies were preserved as archetype *SUMO*s, suggesting subfunctionalization, whereas the third copy served as a hotspot for *SUMO* diversification.
- The Brassicaceae‐specific alpha duplication then caused the duplication of one archetype gamma copy, which, by subfunctionalization, allowed the retention of both *SUMO1* and *SUMO2*. The other archetype gamma copy was simultaneously pseudogenized (*SUMO4/6*). A tandem duplication of *SUMO2* subsequently yielded *SUMO3* in the Brassicaceae crown group. *SUMO3* potentially neofunctionalized in Arabidopsis, but it is lost in many Brassicaceae. Our advanced methodology allows the study of the birth and fixation of other paralogues in plants.

## Introduction

Post‐translational modifications (PTMs) set a reversible mark on proteins, altering their function (van der Veen & Ploegh, 2012). The first polypeptide that was discovered to act as a PTM was ubiquitin (Ub), a highly conserved 76‐residue polypeptide. Ub and ubiquitin‐like modifiers (UBLs) are typified by their β‐grasp fold, which generates a highly stable tertiary structure resistant to environmental perturbations, such as heat (Burroughs *et al*., 2012; Vierstra, 2012; Callis, 2014). There is limited sequence identity between UBL types, yet remarkable sequence conservation is seen for individual UBL types across eukaryotes. For example, Ub is 96% identical between plants, yeast and mammals (Vierstra, 2003).

A prominent UBL type is the Small Ubiquitin‐Like Modifier (SUMO), which is conserved across eukaryotes (Miura & Hasegawa, 2010; Flotho & Melchior, 2013; Jentsch & Psakhye, 2013). Its conjugation is primarily associated with nuclear processes, such as nucleocytoplasmic transport, gene regulation, chromatin remodelling, DNA repair and DNA replication (Miller *et al*., 2010b, 2013; Flotho & Melchior, 2013). SUMO is translated as a precursor that undergoes C‐terminal processing by SUMO proteases (also known as ubiquitin‐like proteases or ULPs). The processing exposes a C‐terminal diglycine (diGly) motif essential for conjugation. Mature SUMO is conjugated to substrates via the E1 SUMO Activating Enzyme dimer (SAE1/2) and the E2 SUMO Conjugating Enzyme (SCE1) (Saracco *et al*., 2007; Castano‐Miquel *et al*., 2013). On conjugation (SUMOylation), an isopeptide bond is formed between the carboxyl terminus of mature SUMO and the acceptor lysine (Lys) side chain. SUMOylation is an essential process, with mutations causing embryonic lethality in mice and the model plant Arabidopsis (*Arabidopsis thaliana*) (Saracco *et al*., 2007; Wang *et al*., 2014). E3 ligases can promote SUMOylation (Flotho & Melchior, 2013). In Arabidopsis, two E3 ligases have been characterized. Loss of the E3 ligase SIZ1 (SAP AND MIZ 1) causes dwarfism, early flowering, altered responses to abiotic stresses and activation of plant immunity (Miura & Hasegawa, 2010; Park *et al*., 2011). By contrast, the E3 ligase High Ploidy2 (HPY2/MMS21) represses endocycle onset in meristems (Huang *et al*., 2009; Ishida *et al*., 2009, 2012). SUMO conjugation is reversible and ULPs catalyse de‐conjugation. Plant ULPs form at least four subgroups that are conserved across angiosperms and function non‐redundantly (Conti *et al*., 2008; Novatchkova *et al*., 2012).

In many eukaryotes, such as budding yeast (*Saccharomyces cerevisiae*), fruit fly (*Drosophila melanogaster*) and the worm *Caenorhabditis elegans,* SUMO is encoded by a single‐copy gene (Flotho & Melchior, 2013). Yet, mammals and Arabidopsis express up to four paralogues. The mammalian paralogues have functionally diversified, modifying distinct but overlapping protein subsets (Citro & Chiocca, 2013). At the sequence level, the mammalian SUMO2 and SUMO3 are very similar (97% sequence identity), whereas SUMO1 only shares 47% sequence identity with SUMO2/3. Functionally, the mammalian SUMO2/3 can form SUMO chains that target their substrates for degradation, whereas SUMO1 cannot (Hay, 2013). SUMO1 and SUMO2/3 also interact with different proteins non‐covalently, as they prefer slightly different SUMO interaction motifs (SIMs) in their partners (Hecker *et al*., 2006; Ghisletti *et al*., 2007; Meulmeester *et al*., 2008). Interestingly, the mammalian *SUMO2* is essential for embryonic development, whereas *SUMO3* is dispensable (Wang *et al*., 2014). This functional difference between *SUMO2* and *SUMO3* appears to be caused by differences in their expression levels, with *SUMO2* being the predominant transcript.

The genome of Arabidopsis encodes eight *SUMO* genes that represent five distinct types (Kurepa *et al*., 2003; Novatchkova *et al*., 2004; Colby *et al*., 2006). Only four of these genes are expressed (Kurepa *et al*., 2003; Saracco *et al*., 2007; Budhiraja *et al*., 2009). From these four genes, *AtSUMO1* and *AtSUMO2* are closely related, sharing 89% protein sequence identity, whereas *AtSUMO3* and *AtSUMO5* share only 48% and 35% identity with *AtSUMO1*, respectively. *AtSUMO1/2* appear to represent the archetype *SUMO*s, as they are the closest homologues of the mammalian SUMO2/3 (with 50% protein identity). Clearly, the archetype *SUMO*s of yeast, mammals and plants have diverged substantially at the protein sequence level since their lineages separated in evolution.

Like their mammalian counterparts, the Arabidopsis *SUMO* paralogues have acquired distinct expression patterns (Van den Burg *et al*., 2010) and biochemical properties. For example, AtSUMO1/2 are better substrates for conjugation than is AtSUMO3 (Castano‐Miquel *et al*., 2011). Second, AtSUMO1/2 can form SUMO chains *in vitro* in the presence of only SAE1/2 and SCE1 (Colby *et al*., 2006; Budhiraja *et al*., 2009). By contrast, chain formation of AtSUMO3 can only be promoted *in vitro* when a truncated form of the SUMO E4 ligase PIAL2 is added (Tomanov *et al*., 2014). Third, the known Arabidopsis ULPs display high (iso)peptidase activity to AtSUMO1/2 conjugates, but low activity to AtSUMO3 conjugates (Chosed *et al*., 2006; Colby *et al*., 2006).

The overexpression of tagged *AtSUMO1* or *AtSUMO2* variants causes the activation of plant immunity, reduced rosette size and altered flowering time (Budhiraja *et al*., 2009; Van den Burg *et al*., 2010). This suggests that enhanced SUMO levels caused by gene duplication of the archetype *SUMO*s potentially result in a fitness cost in plants. A key question is how novel SUMO paralogues have emerged with this evolutionary penalty. Here, we report how the plant *SUMO* family has expanded and diversified in plants, focusing on Brassicaceae (a eudicot family) and Poaceae (a monocot family).

The genome evolution of flowering plants has been massively shaped by palaeoploidy events (Van de Peer *et al*., 2009). For example, one of the largest clades of angiosperms, eudicots, is characterized by an ancient whole‐genome triplication (hereafter called WGT At‐γ) that predates the split of the eudicot clades Asterids, Caryophyllales and Rosids (Tang *et al*., 2008; Dohm *et al*., 2014). Numerous gene duplicates and duplication blocks have been retained from this pan‐eudicot WGT across extant eudicots. Subsequently, two additional whole‐genome duplications (WGDs) (At‐β (88–81 million yr ago (Ma)) and At‐α (47 Ma)) occurred in the Brassicales lineage, which comprises the family Brassicaceae (Vision *et al*., 2000; Hohmann *et al*., 2015). These three palaeopolyploidy events would already have given rise to 12 gene copies in Arabidopsis for any *SUMO* copy present in the ancestral species that underwent At‐γ. Importantly, extensive genome synteny remains from these polyploidy events, both between and within eudicot genomes. We used this genome collinearity (i.e. correlated gene arrangements between genomic regions within and between genomes) to infer ancestry for each of the Brassicaceae *SUMO* genes.

An important model for gene evolution on WGDs is the dosage balance model, based on the notion that retained duplicates tend to be balanced in dosage with each other (Birchler & Veitia, 2007). The dosage balance model *per se* does not address mechanisms of neofunctionalization and, as such, the birth of novel UBL types*,* although the preservation of duplicates is an essential first step for the birth of novel UBL types (Guo *et al*., 2013). In agreement with this model, we reasoned that WGDs will, at first, not imbalance SUMO homeostasis, as the entire (de)conjugation machinery is duplicated. Purifying selection can then be relaxed on one duplicate, allowing it to acquire mutations. Once selection pressure is relaxed, many WGD duplicates are known to be lost. Alternatively, in unique cases, an altered function could be acquired that is beneficial. This could become fixed and then be subject to purifying selection. Our data indicate that this evolutionary model for polyploidy best explains the expansion of the Arabidopsis *SUMO*s, including neofunctionalization, subfunctionalization and the birth and death of novel paralogues.

## Materials and Methods

### Plant SUMO and SUMO‐like (SUL) sequences

Coding sequences of *SUMO* genes were retrieved from whole‐genome and transcriptome assemblies using BLAST searches with the Arabidopsis *SUMO* genes as input sequence. We used Brad (http://brassicadb.org/), Phytozome 10.1 (DOE‐JG, www.phytozome.net) and CoGe (https://genomevolution.org/) as sources (Supporting Information Table S1). The different Brassicaceae *SUMO* homologues were assigned to five groups on the basis of the types identified previously in *A. thaliana* (Kurepa *et al*., 2003), and multiple sequence alignments (MSAs) were made for these orthogroups. The accession numbers of the Brassicaceae *SUMO* genes are listed in Table S2. Support for the expression of different Brassicaceae gene models came from publically deposited transcriptomic data. For several Brassicaceae species*,* we have not included gene IDs in Table S2, as their assemblies lacked gene models. *SUMO* sequences from *Cleome gyandra*,* Boechera stricta*,* Raphanus* species (http://bioinfo.bti.cornell.edu/cgi-bin/radish/index.cgi), *Brassica napus*,* Chorispora bungeana* and *Schrenkiella parvula* (syn. *Eutrema parvulum*) were also retrieved from the NCBI whole‐genome and transcriptome shot gun assemblies. *Brassica oleracea* transcripts were retrieved from an expressed sequence tag (EST) collection (http://brassica.jcvi.org/cgi-bin/brassica/gbrowse.cgi). *Amborella trichopoda* sequences were retrieved from its genome assembly (www.amborella.org/) (Amborella Genome Project, 2013). MSAs were made using Muscle (http://www.ebi.ac.uk/Tools/msa/muscle/). Gene models and alignments were manually corrected using BioEdit (http://www.mbio.ncsu.edu/bioedit/page2.html). Sequences with poor coverage or quality were excluded from further analysis. The SUMO sequence logos were generated with IceLogo (http://iomics.ugent.be/icelogoserver/) (Colaert *et al*., 2009). Thereto, we aligned 153 archetype *SUMO* sequences, including *SUMO* genes from angiosperms, gymnosperms and mosses (*Selaginella moellendorffii*,* Sphagnum fallax*,* Physcomitrella patens* and *Marchantia polymorpha*).

### Gene tree construction

Gene trees were constructed using a maximum likelihood (ML) approach in RaxML (v.7.4.12) with default settings and a GTR + gamma nucleotide model (Stamatakis *et al*., 2008). ML analyses were run on Cipres (http://www.phylo.org/) and the best scoring tree is shown with bootstrap support values at the nodes (Miller *et al*., 2010a). Tree construction for the archetype *SUMO* genes from eudicots was based on 109 aligned sequences with a length of 384 nucleotides (259 differential patterns); the species used are indicated in Table S1. The *SUMO5* tree was reconstructed using 41 aligned sequences (nucleotide length of 241, with 209 differential patterns): *Tarenaya hassleriana*,* Aethionema arabicum*,* Arabidopsis thaliana*,* A. lyrata*,* A. halleri*,* A. arenosa*,* Boechera stricta*,* Capsella rubella*,* Camelina sativa*,* Chorispora bungeana*,* Brassica rapa*,* B. oleracea*,* Raphanus raphanistrum*,* R. sativus*,* Eutrema salsugineum*,* Arabis alpina*,* Leavenworthia alabamica*,* Neslia paniculata*,* Schrenkiella parvula* and *Sisymbrium irio*. We removed, in this case, poorly aligned regions from both the N‐ and C‐termini. The Brassicaceae *SUMO1/2* tree was inferred using 49 aligned sequences (351 nucleotides and 267 differential patterns). The pruned Brassicaceae phylogeny tree was based on published data (Couvreur *et al*., 2010; Franzke *et al*., 2011; Haudry *et al*., 2013; Moghe *et al*., 2014).

### Clustering analysis of the syntenic gene pairs

For the synteny‐based approach, we retrieved syntenic gene pairs (between and within genomes) using genome collinearity. Gene pairs were retrieved from the Plant Genome Duplication Database (PGDD) (http://chibba.agtec.uga.edu/duplication/) (Lee *et al*., 2013). The accession numbers of the dicot *SUMO/SUL* genes obtained are listed in Table S3. These gene pairs were represented in a network in Cytoscape (Cline *et al*., 2007) using the Files ‘Organic*’* network lay‐out. The network representation was manually optimized to depict the three major gene *SUMO/SUL* clusters and to highlight their interaction with the Brassicaceae *SUMO* paralogues. Support for the network organization is based on the number of syntenic gene pairs between twinned genomic blocks and the scores provided for these blocks by PGDD. Edge thickness represents log(number of anchors), but a similar representation was obtained with log(score). As *Tarenaya hassleriana* (syn. *Cleome spinosa*) and *Aethionema arabicum* are not represented in PGDD, we performed, for these species, separate GeVo analyses in CoGe (https://genomevolution.org/coge/GEvo.pl) to obtain syntenic paralogous relationships between the *SUMO* genes from *T. hassleriana, A. arabicum,* Arabidopsis and eucalyptus (*Eucalyptus grandis*).

### *SUMO* gene evolution in the Arabidopsis population

Sequence conservation of the Arabidopsis *SUMO* genes was assessed using the data from the 444 Arabidopsis accessions sequenced (http://signal.salk.edu/atg1001/3.0/gebrowser.php). We determined the percentage of accessions that contained an amino acid other than the prevalent residue for each position for the eight Arabidopsis SUMO paralogues. Subsequently, we aligned the SUMO paralogues in a protein MSA. We then generated a heat map of the MSA depicting the percentage of accessions (%) containing a different residue at a particular position in the MSA for each position in the alignment. The heat map was generated in R (http://www.r-project.org) using Heatmap.2 (gplots package) with the grey2yellow colour key.

## Results

**Figure 1:**
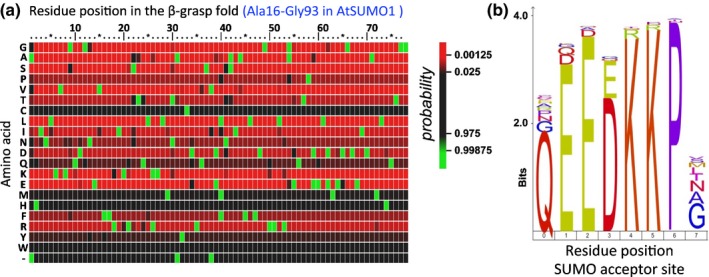
The sequences of the β‐grasp fold and the Small Ubiquitin‐Like Modifier (SUMO) acceptor motif are conserved across land plants. (a) A heat map diagram of the protein sequence alignment of archetype SUMOs from land plants, demonstrating extreme sequence conservation across the entire β‐grasp fold; of 77 positions in the β‐grasp fold, 69 positions (90%) are nearly invariant and, for the other eight positions, we observed predominantly substitutions of the presumed ancestral state for a similar residue: 25[KR], 29[TN], 39[MFL], 41[AS], 53[GA] and 38/58/61[DE]. The colour indicates the probability of a certain amino acid at that position. (b) IceLogo of the SUMO acceptor site in the N‐terminus of SUMO shows that the motif is strictly conserved in land plants. The same set of sequences is used as in (a).

To reconstruct the evolution of the Arabidopsis *SUMO* paralogues, we searched for homologues of the five Arabidopsis SUMO types (*AtSUMO1/2*,* AtSUMO3*,* AtSUMO4/6*,* AtSUMO5* and *AtSUMO7/8*) in plant genome assemblies. We always identified at least one close homologue of *AtSUMO1/2* in each plant genome analysed, but close homologues were absent for the other SUMO types outside the Brassicaceae family. We only found one exception to this rule, that is, we found a *SUMO5* orthologue (Th15853) in *T. hassleriana;* this species belongs to the closest sister family of Brassicaceae: Cleomaceae (Cheng *et al*., 2013). This implies that: (1) the Arabidopsis *SUMO* paralogues other than *SUMO1/2* first emerged in a common ancestor of Brassicaceae/Cleomaceae; and (2) *SUMO1/2* represents the archetype *SUMO* in plants. The protein sequence of these archetype *SUMO* homologues proved to be extremely conserved from mosses to angiosperms, specifically across the β‐grasp fold (Ala16–Gly93 for AtSUMO1) (Fig. 1a). C‐terminal to the diGly motif, the sequence is not conserved, whereas N‐terminal to the β‐grasp fold, a second motif was found to be conserved (Fig. 1b). This six‐residue motif probably acts as an internal SUMO acceptor site (QEE[D/E]KK*P, with * indicating the acceptor Lys); at least *in vitro* this Lys acts as a SUMO acceptor site (Colby *et al*., 2006). This acceptor motif is retained from mosses (*P*. *patens*,* S. fallax* and *M. polymorpha*) to angiosperms with a variant motif in *S. moellendorffii* (DVKPEKKP). Mosses like *P. patens* split *c.* 500 Ma from the lineage, leading to angiosperms (Hedges *et al*., 2015). Combined, this indicates that the archetype SUMO protein is extremely conserved in land plants and that SUMO chain formation is potentially as well conserved.

### Only one of two ancient archetype SUMO genes of eudicots is retained in Brassicaceae

Subsequently, we examined the moment of birth of the Arabidopsis *SUMO1* and *SUMO2* genes. Interestingly, we only found one *SUMO* gene (ID: AmTr_v1.0_scaffold00228: 122 523–131 678 bp) in the genome of the basal angiosperm *Amborella trichopoda* (Amborella Genome Project, 2013)*. Amborella trichopoda* forms an outgroup to most other extant angiosperms (with an estimated split at *c*. 147 Ma), including the monocots and dicots. By contrast, most monocot and dicot genomes analysed contained extra *SUMO* gene copies (Table S1). This indicates that a common ancestor of the angiosperms potentially carried a single *SUMO* gene and that, during monocot and eudicot radiation, this ancestral gene was duplicated.

**Figure 2:**
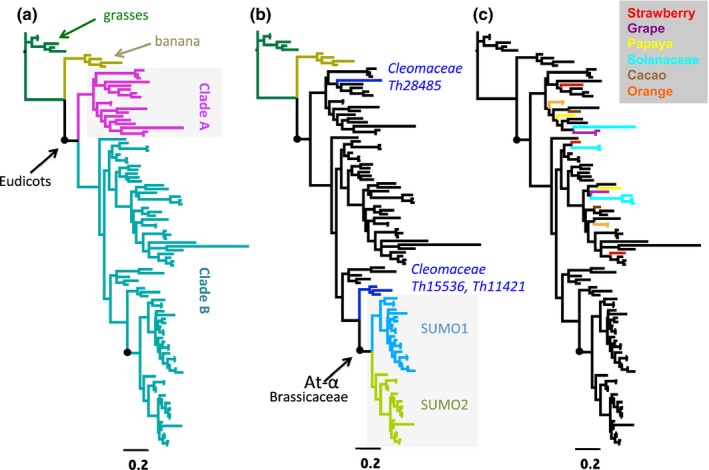
In a common ancestor of eudicots, the archetype *Small Ubiquitin‐Like Modifier* (*SUMO*) gene was duplicated with both copies being broadly retained, except for Brassicaceae. (a) Gene tree diagram of eudicot *SUMO* genes demonstrates that they split into two distinct clades (clades A and B). *SUMO* homologues from grasses (Poaceae) and banana were used as outgroup. (b) Same tree as in (a). Brassicaceae *SUMO1* and *SUMO2* cluster uniquely with clade B, whereas archetype *SUMOs* from *Tarenaya hassleriana* split over both clades. *Tarenaya hassleriana* belongs to the nearest sister family of Brassicaceae: Cleomaceae. The indicated gene IDs come from the *T. hassleriana* genome assembly. The Brassicaceae‐specific At‐α polyploidy event is indicated on the branch. (c) Same tree as in (a). Both clades first emerged in a common ancestor of eudicots, as they are both represented in Asterids (Solanaceae species tomato and potato (cyan)) and Eurosids (strawberry (red), grape vine (purple), papaya (yellow), cacao (brown) and sweet orange (orange)).

Based on this notion, we constructed an ML gene tree for a set of Brassicaceae *SUMO1/2* genes and a core set of archetype *SUMO* genes from eudicot genomes other than Brassicaceae; this set included sequences from both Rosids and Asterids (Fig. 2). As outgroup for this tree, we used *SUMO* homologues of monocots (grasses and banana (*Musa acuminata*)). The gene tree revealed the existence of two major SUMO clades in eudicots (Fig. 2a). SUMO proteins in Clade A are recognizable by a variable stretch of glycines, which starts at position + 4 from the translational start; this stretch of glycines is absent in the Clade B *SUMO* genes. Importantly, *AtSUMO1* and *AtSUMO2* both grouped with Clade B. In fact, all Brassicaceae *SUMO* genes grouped with Clade B, whereas the archetype *SUMO* genes from *T. hassleriana* split over both clades (Fig. 2b). This indicates that Clade A was recently lost in Brassicaceae since the split with Cleomaceae (*c*. 52 Ma). In agreement, we found that both clades are represented in the genome of papaya (*Carica papaya*). Papaya represents a basal Brassicales that separated before the At‐β WGD. Also, in the genomes of sweet orange (*Citrus *×* sinensis*) and cacao (*Theobroma cacao*), both SUMO clades are represented (Fig. 2c). Sweet orange and cacao belong to sister orders of Brassicales, namely Sapindales and Malvales (Hohmann *et al*., 2015; Magallon *et al*., 2015). Both clades are also represented in the genomes of eucalyptus and grape (*Vitis vinifera*); both of these species belong to basal Eurosid lineages. In fact, both SUMO clades were also present in Asterids, for example, potato (*Solanum tuberosum*), tomato (*S. lycopersicum*) and kiwi (*Actinidia chinensis*). Combined, this means that at least two archetype *SUMO* genes have coexisted for > 125 million yr in many eudicots, but that one copy was lost specifically in a common ancestor of the Brassicaceae family.

### The Arabidopsis SUMO1 and SUMO2 genes are recent At‐α duplicates

We found that orthologues of *AtSUMO1* and *AtSUMO2* are present in each Brassicaceae genome analysed, including *Aethionema arabicum*. This species represents a basal Brassicaceae lineage that split before radiation of the Brassicaceae crown group (*c*. 32 Ma) (Beilstein *et al*., 2010; Kagale *et al*., 2014; Hohmann *et al*., 2015). We noted little protein sequence variation between *AtSUMO1/2* and their Brassicaceae orthologues. The *AtSUMO1/2* genes are syntenic paralogues, that is, they are located in a duplicated genomic block consisting of 90 syntenic genes (homologous gene pairs that are arranged in a related order on both genomic blocks). This duplication block was present in all Brassicaceae analysed and carries an At‐α signature, that is, the mean synonymous substitution value per synonymous site (*K*s) of this duplication block (mean *K*s ± SD = 0.91 ± 0.32; 90 gene anchors) corresponds to the mean *K*s of the At‐α duplication blocks combined (*K*s = 0.77) and not to the mean *K*s of the At‐β blocks (*K*s = 2.05) (Fig. S1) (Kagale *et al*., 2014). At‐α is absent in *T. hassleriana* (family Cleomaceae). Instead, *T. hassleriana* has experienced its own WGT (Th‐α) (Cheng *et al*., 2013). In agreement with this, we see that the Clade B *SUMO* genes of *T. hassleriana* form a separate branch (Th15536*,* Th11421) in the ML tree, which is positioned sister to the Brassicaceae *SUMO1* and *SUMO2* branches (Fig. 2b; grey box). From this analysis, we conclude that this *AtSUMO1/2* duplication emerged as a result of At‐α and that both genes have been retained across Brassicaceae ever since.

### *SUMO5* appears to have neofunctionalized in Brassicaceae

**Figure 3:**
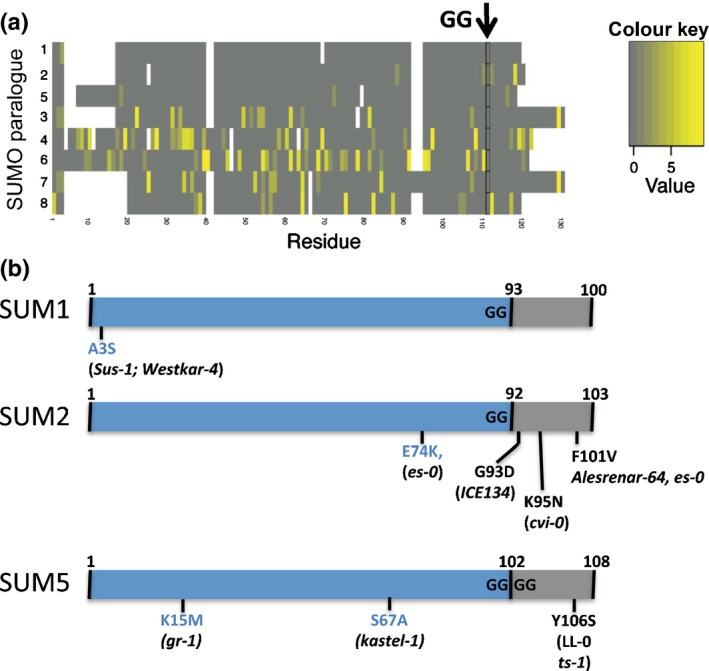
The protein sequences of AtSUMO1, AtSUMO2 and AtSUMO5 are highly conserved in the Arabidopsis population. (a) Heat map diagram of a protein sequence alignment displaying the percentage of amino acid substitutions per residue (grey to yellow) for the different Arabidopsis Small Ubiquitin‐Like Modifier (SUMO) paralogues. *AtSUMO1*,* AtSUMO2* and *AtSUMO5* have a few dominant alleles in the population with few accessions carrying an amino acid substitution, whereas, for the other paralogues, many alleles exist in the population with numerous substitutions scattered over the encoded proteins. The diglycine (diGly) motif is indicated (black arrow). The white interruptions indicate gaps in the alignment. (b) Diagram of the three conserved Arabidopsis SUMO paralogues with the substitutions found in the different accessions indicated. Blue, the mature protein; grey, the C‐terminal part removed during processing.

We also analysed the sequence variation of the eight Arabidopsis *SUMO* paralogues in 444 accessions. We found a substantial number of alleles that contained non‐synonymous mutations for the four pseudogenes (*AtSUMO4, AtSUMO6, AtSUMO7* and *AtSUMO8*). Similarly, many coding mutations were found for *AtSUMO3,* affecting its entire protein coding sequence (Fig. 3a). However, *AtSUMO1* and *AtSUMO2,* but also *AtSUMO5*, were practically invariant at the protein level in the Arabidopsis population. For *AtSUMO2,* one non‐synonymous mutation was found that was present in 36 accessions, affecting the processed C‐terminal tail (F101V). Other mutations, which affect the mature AtSUMO2 protein, were only found in unique accessions (Fig. 3b). For *AtSUMO1*, only two accessions carried a non‐synonymous mutation (A3S).

**Figure 4:**
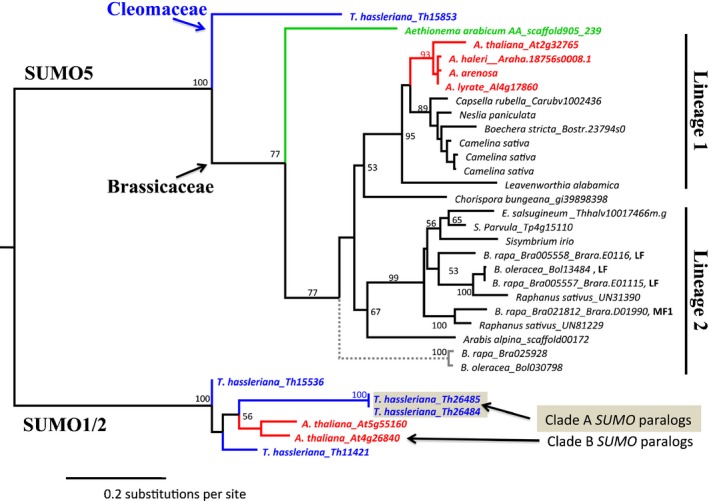
SUMO5 emerged before the split of the sister families Brassicaceae and Cleomaceae, as exemplified by *Tarenaya hassleriana* Th15853. The gene tree of the *SUMO5* family shows that the family is less conserved than *SUMO1* or *SUMO2*. Syntenic paralogues of *SUMO5,* which emerged from a *Brassica‐*specific whole‐genome triplication (WGT) event (Br‐α), are indicated by LF and MF1; Bra005557 + Bra005558 represent a tandem duplication. The genus *Brassica* also contains a putative *SUMO5* pseudogene (ψ) that lacks the diglycine (diGly) motif (Bra025928, Bol03070798). As outgroup, we used *SUMO1* and *SUMO2* homologues of Arabidopsis and *T. hassleriana*; bootstrap support values are indicated for the different branches.

In the case of *AtSUMO5,* four accessions contained an allele that encoded an amino acid substitution compared with its sequence in the accession Col‐0. Orthologues of *SUMO5* are conserved across Brassicaceae, including *A. arabicum*, but are more divergent than the *SUMO1/*2 orthologues (Fig. 4; based on branch lengths). Gene expression data (EST and whole transcriptome data) indicate that many *SUMO5* orthologues are expressed (Table S2). Several Brassicaceae *SUMO5* transcripts (Bra005558, Bra021812, Thhalv10015519) already encode a mature SUMO protein with three glycines exposed at the C‐terminus, indicating that processing would not be needed for these variants. Importantly, there is a close homologue of *SUMO5* in *T. hassleriana* (Th15853), but not in the more basal Brassicales papaya. *SUMO5* must therefore have evolved prior, but relatively close to, the split of Brassicaceae and Cleomaceae (Kagale *et al*., 2014). Since then, *SUMO5* has potentially neofunctionalized, but future studies should reveal its function.

### Identification of three ancient *SUMO* gene lineages in eudicots

*ThSUMO5* resides in a genomic region that is syntenic with *AtSUMO5*, sharing 20 collinear genes (Fig. S2). This genomic region is also syntenic with a genomic region in eucalyptus, but, instead, eucalyptus contains a divergent *SUL* gene (Eugr.H0049, E.grandis_v1_0.046213m) at the corresponding position (Table S3). Eucalyptus belongs to the order Myrtales, a lineage that is sister to the Eurosids (Myburg *et al*., 2014). The split of Myrtales and Eurosids is currently estimated to have been at *c*. 135–110 Ma, which implies that *SUMO5* evolved from a *SUMO* paralogue that first emerged before eudicot radiation.

**Figure 5:**
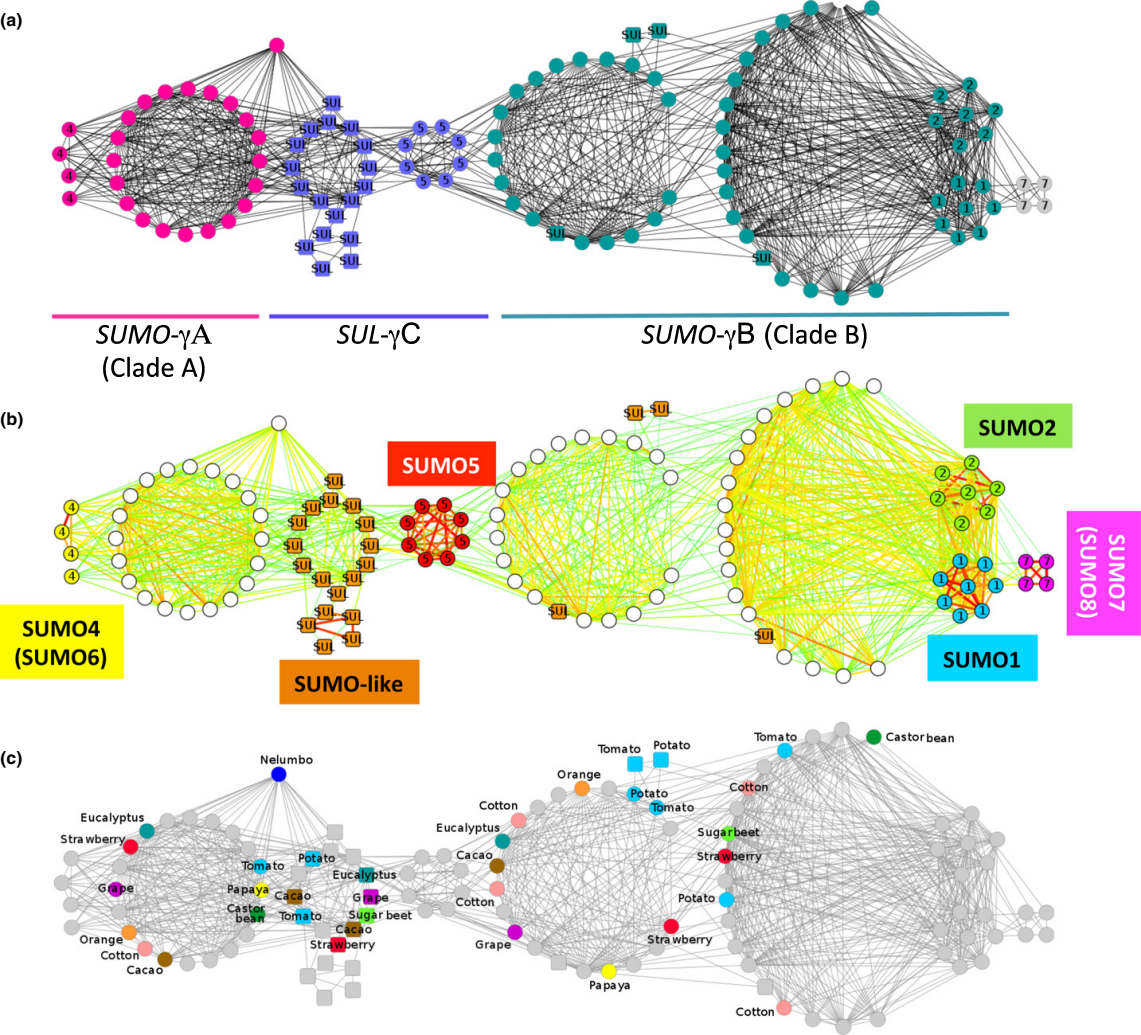
The Brassicaceae *Small Ubiquitin‐Like Modifiers* (*SUMO*s) evolved from three ancient syntenic paralogues that probably emerged from the pan‐eudicot whole‐genome triplication (WGT) (At‐γ). (a) Network representation of genome collinearity between *SUMO* gene pairs found within and between eudicot genomes. Two principal clusters contain the archetype *SUMOs*:*SUMO*‐γA and *SUMO*‐γB (purple and cyan circular nodes). These two clusters are not interconnected, but show weak synteny with a third cluster comprising *SUMO5* from Brassicaceae and *SUMO‐like* genes from non‐Brassicaceae species (SUL‐γC; blue square nodes). The edges represent synteny between genomic regions that surround the connected genes; the numbers in the nodes refer to the Brassicaceae *SUMO* paralogues (1: *AtSUMO1*, etc.). (b) Same network as in (a), except that the Brassicaceae *SUMO* paralogues are indicated and the line width and colour of the edges reflect the number of syntenic genes per gene pair (green to red, low to high number of syntenic genes). Brassicaceae *SUMO1* (blue) and *SUMO2* (green) cluster with *SUMO*‐γB, whereas *SUMO4* (yellow) groups with *SUMO*‐γA. *SUMO7* (magenta) is best connected to *SUMO1,* whereas *SUMO5* (red) is linked to both the *SUMO*‐γB and *SUL*‐γC genomic regions. (c) Same network as in (a), but the nodes are coloured per species. *SUMO* genes from Asterids, Rosids and Caryophyllales have representatives in each of the principal three clusters (*SUMO*‐γA, *SUMO*‐γB and *SUL*‐γC). The basal dicot sacred lotus (*Nelumbo nucifera*) is represented by a single *SUMO* gene, which shows synteny with both *SUMO*‐γA and *SUL*‐γC clusters, but not *SUMO*‐γB.

To further date the birth of *SUMO5*, we screened for syntenic pairs of *SUMO* and *SUL* genes (using PGDD) and performed a network analysis on the gene pairs obtained using Cytoscape (Fig. 5; Table S3). This network depicts *SUMO/SUL* genes (nodes) that are connected by edges, which represent genome collinearity between gene pairs. The analysis revealed three major interconnected clusters of collinear genes. The two aforementioned archetype SUMO clades (Fig. 2) split perfectly over two of the three clusters, with no evidence for collinearity between them (Fig. 5). As *SUMO* genes from both Rosid and Asterid species are represented in both clusters, their ancestral genes must have emerged before the split of Rosids and Asterids. For example, the Rosids strawberry (*Fragaria vesca*), eucalyptus and grape have members in both clusters. As these three species have not undergone any additional polyploidization since At‐γ (Murat *et al*., 2012), these two *SUMO* clusters probably represent At‐γ syntenic paralogues or evolved shortly after by a gene transposition duplication event. Thereafter, homologues of both genes have been retained in many eudicots, but not in Brassicaceae (Figs 2, 5b).

The third cluster identified comprises a set of divergent *SUL* sequences. We estimate the birth of this third cluster also at/or around At‐γ, because Rosids, Asterids and Caryophyllales (sugar beet (*Beta vulgaris*)) have members in this third cluster. The moment of birth of these three clusters is supported by the basal dicot sacred lotus (*Nelumbo nucifera*), which diverged from eudicots before At‐γ (Ming *et al*., 2013). Sacred lotus contains an archetype *SUMO* (NNU_022372‐RA) that shares collinearity with both the *SUMO‐*γA and *SUL‐*γC clusters, but not with the *SUMO‐*γB cluster. In conclusion, we found three ancient *SUMO/SUL* gene lineages that appear to represent At‐γ syntenic paralogues: *SUMO*‐γA, *SUMO‐*γB and *SUL*‐γC.

### *SUMO5* resides in a genomic region that acts as a hotspot for SUMO paralogue evolution in eudicots

As the *SUMO5* orthologues show weak synteny to both the *SUMO*‐γB and *SUL‐*γC clusters (and no synteny with the *SUMO‐*γA cluster), we examined in more detail to which cluster *SUMO5* belongs. Close inspection of the synteny between *T. hassleriana SUMO5* (Th15853), *AtSUMO5* (At2g32765) and the eucalyptus *SUL* gene Eucgr.H00049 (*SUL‐*γC) indicates that *SUMO5* most probably emerged from *SUL‐*γC and not *SUMO*‐γB (Fig. S2). In agreement, the genomic block that surrounds *A. lyrata SUMO5* is better connected with the *SUL‐*γC genes from the basal eudicots eucalyptus and grape vine than with the *SUMO*‐γB genes from these same species (Table S4). Another argument that *SUMO5* emerged from *SUL‐*γC is that the genes in this cluster appear to diverge, that is, the *SUL‐*γC sequences do not form a gene tree that is consistent with their species tree. By contrast, the two other clusters contain primarily close homologues of the archetype *SUMO* and their sequences diverge little. We therefore propose that *SUMO5* most probably emerged from a *SUL‐*γC predecessor.

### Origin of the Brassicaceae SUMO4 and SUMO7 orthogroups

The four *Arabidopsis SUMO* pseudogenes are arranged as two tandem duplications (TDs), that is, *AtSUMO4*::*AtSUMO6* (At5g48700, At5g48710) and *AtSUMO7*::*AtSUMO8* (At5g5‐5855, At5g55856) (Kurepa *et al*., 2003). However, in other Brassicaceae – including the genus *Arabidopsis* (*A. halleri*,* A. arenosa* and *A. lyrata*), they are present as singletons at syntenic scaffolds. Therefore, both TDs probably occurred during *A. thaliana* speciation. Interestingly, *AtSUMO4* shares collinearity with *SUMO‐*γA genes, including eucalyptus Eucgr.H00789 and the *T. hassleriana* TD gene pair Th26484 and Th26485 (Figs 5, S3; Table S3). For example, we found 25 collinear genes between Arabidopsis and *T. hassleriana*. This indicates that *AtSUMO4* and Th26484::Th26485 are syntenic orthologues. *SUMO4* is also present in *A. arabicum* and other Brassicaceae (Table S2). This means that *AtSUMO4* must have emerged in a common ancestor of Brassicaceae after the split of Cleomaceae, and that it probably evolved from a *SUMO‐*γA descendant.

Related to this, we noted that Brassicaceae *SUMO7* shows collinearity with both Brassicaceae *SUMO1/2*, but not with *SUMO4* or *SUMO5* (Fig. 5b). The average *K*s between the *SUMO7* and *SUMO1/2* genomic regions is *c*. 1.0–1.1. This is more than expected for At‐α (*K*s = 0.77), but less than expected for At‐β (*K*s = 2.05) (Kagale *et al*., 2014). This means that *SUMO7* probably emerged from a segmental duplication of *SUMO1* or *SUMO2*. The birth of *SUMO7* appears to coincide with At‐α, as *A. arabicum* contains a putative orthologue (Table S2), but *T. hassleriana* does not. *SUMO7* is also present in the Brassicaceae lineage II (including Brassica) (Table S2). In two Brassica species, *SUMO7* is present as a misannotated singleton. In *B. rapa,* a homologous sequence is present in the intergenic region between Bra00287070 and Bra00287071, whereas, in *B. oleracea*, the corresponding gene is misannotated (Bol006236). Certain *SUMO4* and *SUMO7* orthologues have retained their diGly motif, whilst transcripts were also reported for *SUMO4* in *B. oleracea* and *C. bungeana*, whereas, for *SUMO7*, a transcript was reported for *C. rubella*. This could mean that certain *SUMO4* and *SUMO7* orthologues still encode functional proteins.

### *SUMO3* emerged from a *SUMO2* TD after divergence of *A. arabicum*, but before radiation of the Brassicaceae crown group

**Figure 6:**
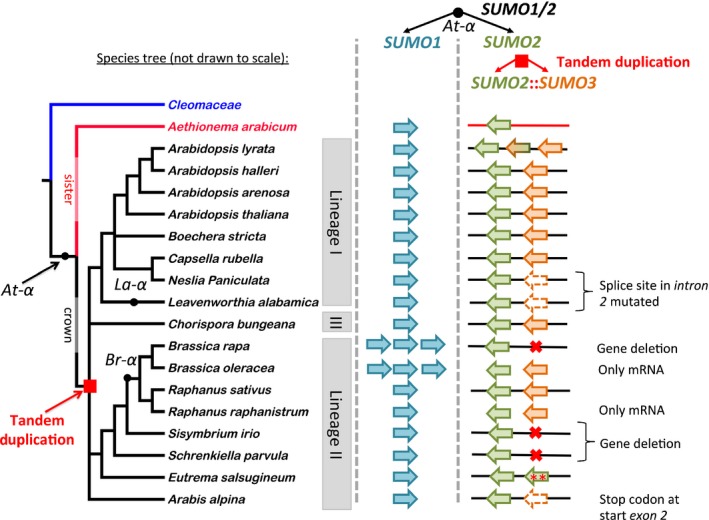
*SUMO3* emerged before radiation of the Brassicaceae crown species. *SUMO1* (light blue arrow) and *SUMO2* (green arrow) are At‐α syntenic paralogues (black dot) based on collinearity between their genomic regions. Subsequently, a tandem duplication (TD) (red square) occurred at the *SUMO2* locus (green and orange arrows) before radiation of the Brassicaceae crown group into three lineages. The phylogenetic tree represents a pruned Brassicaceae family tree with Cleomaceae (*Tarenaya hassleriana*) as outgroup (blue) and *Aethionema arabicum* (red) at the base of the Brassicaceae family tree. Various Brassicaceae lack a functional *SUMO3* gene as a result of gene deletion, conversion or mutations that affect the reading frame. The *Arabidopsis lyrata* genome contains, in addition, a hybrid *SUMO2‐3* gene.

Similar to the two aforementioned Arabidopsis *SUMO* pseudogenes, *AtSUMO2* and *AtSUMO3* represent a TD (Fig. 6). This TD is present in many, but not all, Brassicaceae genomes. For example, this duplication is absent in the basal Brassicaceae *A. arabicum*. In *E. salsugineum*, two *EsSUMO2* copies are present in tandem at this locus, suggesting a recent gene conversion of *SUMO3*. This is supported by the increased branch length of one of the two *EsSUMO2* genes (*; Thhalv10015081) (Fig. S4). In the Brassica species *B. rapa* and *B. oleracea, SUMO3* appears to be deleted from all three subgenomes (Brassica emerged from a recent ancestral hexaploid *c*. 20–24 Ma (Br‐α)), that is, BLAST searches did not reveal any homology to *AtSUMO3*. Nonetheless, *SUMO3* transcripts were reported for *B. oleracea* (asmbl_13151; http://brassica.jcvi.org/cgi-bin/brassica/index.cgi) and *B. napus* (NCBI ES966440.1). The latter species is an allotetraploid of *B. rapa* and *B. oleracea*. Possibly, a single *SUMO3* copy has been retained in some, but not all, *Brassica* cultivars. In support of this, a *SUMO3* copy is retained in the genus *Raphanus,* which shares the Br‐α WGT and only recently diverged from the genus *Brassica* (5–16 Ma) (Moghe *et al*., 2014; Hohmann *et al*., 2015).

Importantly, the ML gene tree of *SUMO1, SUMO2* and *SUMO3* combined indicates that *SUMO2* from *A. arabicum* forms a branch that is basal to the *SUMO2* and *SUMO3* clades in the gene tree. The most parsimonious explanation is that *SUMO3* emerged from a TD of *SUMO2* after the split of *A. arabicum* (*c*. 32 Ma), but before radiation of the Brassicaceae crown group (Hohmann *et al*., 2015). On duplication, one duplicate appears to have rapidly diversified, yielding *SUMO3*, whereas the other duplicate remained nearly unchanged (*SUMO2*). The crown group is subdivided into three lineages. The split between lineage I–III and II is currently estimated at *c*. 23 Ma (Hohmann *et al*., 2015). *SUMO3* orthologues are widely found and expressed in lineage I. However, in lineage II, *SUMO3* is often pseudogenized (via early stop codons and mutation of intron‐splice sites), deleted or subject to gene conversion. As the two genes have co‐evolved, it is evident that *SUMO2* is under purifying selection, whereas *SUMO3* appears to be non‐essential in many Brassicaceae.

### Birth of grass‐specific *SUMO* paralogues

In the genomes of grasses (Poaceae), we identified three distinct *SUMO/SUL* loci. Two loci are genetically linked on chromosome 2 of *Brachypodium distachyon*. They represent an archetype *SUMO* (Bradi2g58830) and, 2.7 Mb upstream, an uncharacterized grass‐specific *SUMO* paralogue (Bradi2g55140), hereafter called *Grass SUMO‐Like 1* (*GSUL1*). Functional data are lacking for this *GSUL1*, but *GSUL1* from sorghum encodes a conjugation‐deficient variant, indicating that it cannot act as PTM. In rice and maize, an orthologue of *GSUL1* is missing, whereas the archetype *SUMO* is represented by a TD. Interestingly, this grass locus with archetype *SUMO* genes is related to the eudicot *SUMO*‐γA cluster, as the *SUMO* genes from banana (*M. acuminata* GSMUA_Achr8G00860::70 TD) and oil palm (*Elaeis guineensis* p5_sc00157.V1.gene38) show synteny with both the eudicot *SUMO‐*γA cluster and this grass locus (based on PGDD). Representatives of this grass locus are Bradi2g58830, maize (*Zea mays*) GRMZM2G053898, sorghum (*Sorghum bicolor*) Sobic.003g402600 and rice (*Oryza sativa*) Os01g68940. On the other hand, we found no synteny with the eudicot *SUMO*‐γB cluster, which supports our notion that *SUMO*‐γB only first appeared after the At‐γ WGT.

**Figure 7:**
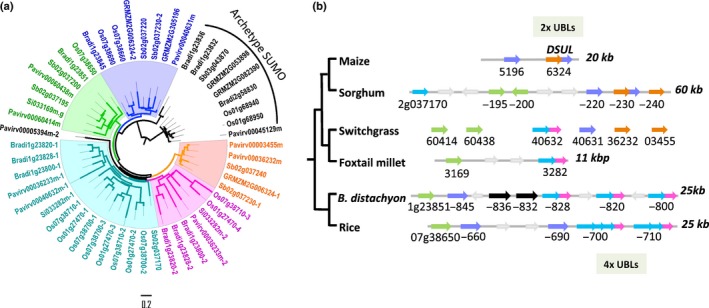
The locus in grasses that is homologous to the maize *DiSUMO‐like* (*DSUL*) locus acts as a ‘hotspot’ for *SUMO‐like* gene evolution, including the formation of concatemers by head‐to‐tail fusions of ubiquitin‐like modifier (UBL) domains. (a) A gene tree based on the single UBL domains from GSUL2 isoforms and UBL concatemers found at the *DSUL* locus in grasses. The UBL domains form five distinct clades. The UBL domains of *DSUL* group into different clades than the UBL domains of the rice concatemers. This implies that the gene fusions have independently occurred in ancestors of maize and rice. As outgroup, we used the archetype *SUMO* gene from grasses; bootstrap support values are shown in Supporting Information Fig. S5. (b) Schematic representation of the *GSUL2/DSUL* loci in grasses, indicating the different gene fusions and TD events found at this locus based on the maximum likelihood (ML) tree. The colours of the UBL domains (arrows) reflect the different clades seen in the ML tree. For orientation, the different gene identifiers are indicated.

The third locus represents a hypervariable multi‐gene locus that contains a second grass‐specific *SUL* gene, hereafter called *GSUL2*. The locus is composed of a variable number of *GSUL2* genes in different grass genomes, suggesting active gene duplications and rearrangements (Fig. 7). It is not only composed of genes with a single UBL domain, but also harbours concatemers of UBL domains. One such concatemer has been characterized previously: the maize gene *DiSUMO‐like* (*DSUL*, GRMZM2G006324; Srilunchang *et al*., 2010). Some of these *GSUL2* genes encode conjugation‐deficient SUL proteins lacking a diGly motif, for example, Sobic.002g350100 from sorghum. Likewise, in several cases, the concatemers have lost their internal and/or C‐terminal diGly motifs, meaning that they cannot be proteolytically cleaved in conjugation‐competent single or multimeric GSUL2 units. A gene tree based on the individual UBL domains of the *GSUL2* homologues, *DSUL* and other concatemers exposed that *DSUL* represents a gene fusion of two progenitor *GSUL2* genes that group with two different clades in the gene tree (Figs 7a, S5). Moreover, the UBL domains from the rice concatemers also branch over two clades, but these two clades with rice UBLs do not overlap with the two DSUL clades (Fig. 7). This implies that the maize *DSUL* and two rice concatemers, Os07g38700, Os07g38710, emerged from independent gene fusion events. In conclusion, grasses contain an additional *SUL* gene cluster that actively evolves via TDs in combination with gene fusions.

## Discussion

**Figure 8:**
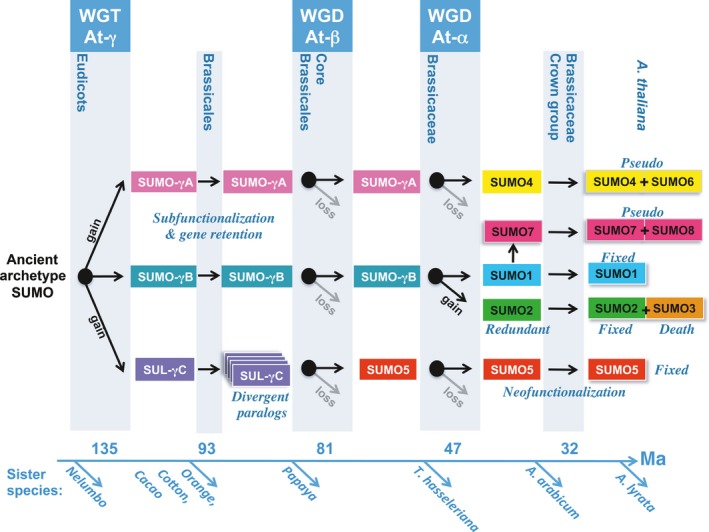
Model for the expansion and diversification of the Brassicaceae Small Ubiquitin‐Like Modifier (SUMO) gene family. The ancestral archetype *SUMO* gene was triplicated by the eudicot‐specific hexaploidy event At‐γ (gain). Two duplicates were broadly retained as archetype SUMOs (*SUMO‐*γA and *SUMO*‐γB) in eudicots, possibly as a result of subfunctionalization, whereas the third copy diversified shortly after (*SUL*‐γC) and became fixed as *SUMO5* before the split of the Cleomaceae (*Tarenaya hassleriana*) and Brassicaceae families. Subsequently, the At‐α whole‐genome duplication (WGD) caused duplication of *SUMO‐*γB yielding Brassicaceae *SUMO1/2*. A subsequent tandem duplication of *SUMO2* in a common ancestor of the Brassicaceae crown group provided *SUMO3* (gain). *SUMO3* is frequently lost by gene deletion, conversion or other mutations, whereas *SUMO2* is 100% retained. Around this time, the *SUMO‐*γA homologue was pseudogenized in a recent Brassicaceae ancestor giving *SUMO4*. Likewise, a segmental duplication of *SUMO1* or *SUMO2* appears to have yielded the *SUMO7* pseudogene (gain) in a recent Brassicaceae ancestor. The positions of the three most recent Arabidopsis palaeo‐polyploidy events are indicated along the top of the *x*‐axis (bottom). Informative sister species for this model are indicated at the bottom along the *x*‐axis. Ma, million years ago.

We examined the evolution and diversification of the *SUMO* family across angiosperms, and in greater detail in Brassicaceae and Poaceae, to understand the dynamics and evolution of novel UBLs. Expansion and divergence of the *SUMO* family is impacted by WGDs and TDs. The *SUMO* landscape is extensively shaped by the pan‐eudicot At‐γ WGT. From this WGT, three *SUMO* loci (*SUMO*‐γA, *SUMO*‐γB and *SUL*‐γC) are preserved across eudicots, of which two loci encode archetype *SUMOs* (*SUMO*‐γA, *SUMO*‐γB; Fig. 8). These *SUMOs* have remained nearly identical, suggesting that the ancestral palaeo‐eudicot *SUMO* genes subfunctionalized in their expression pattern or gene dosage. Importantly, the genes that belong to these two syntenic clusters split perfectly over two distinct branches in the gene tree without cases of gene conversion (Fig. 2; Table S3). We therefore rule out that they co‐evolved by concerted evolution, as reported for ubiquitin (Nei *et al*., 2000). From the same period, a third locus emerged that represents a diversifying orthogroup, represented by *SUMO5* in Brassicaceae (Fig. 4; Table S3). Genome collinearity indicated that a *SUMO5* ancestor (*SUL*‐γC) first emerged close to the At‐γ event (Figs 5, 8) – a moment in which the entire SUMO machinery was triplicated. Homologues derived from this ancestral *SUL*‐γC locus are now highly divergent between eudicot families. Hence, this genomic region acts as a hotspot for *SUMO* paralogue evolution. Similarly, grasses also contain a locus that acts as a hotspot for SUL evolution (Fig. 7); this locus contains both single UBL‐domain *SUL* genes and genes encoding concatemers of UBL domains.

We found that the *AtSUMO5* sequence is nearly invariant in Arabidopsis*,* which suggests that it has neofunctionalized. In agreement, the overexpression of mature AtSUMO5 resulted in its conjugation to unknown plant proteins (Budhiraja *et al*., 2009), indicating that it can act as PTM. Brassicaceae SUMO5 homologues have retained their diGly motif for > 52 million yr, whereas homologues of the ‘younger’ SUMO4 have frequently lost their diGly motif. Certain *SUL*‐γC homologues have also retained their diGly motif, suggesting that they could act as PTMs (Table S3). Biochemically, SUMO5 appears to have diverged from the canonical conjugation pathway. For example, Arabidopsis SAE1/2 and SCE2 can attach AtSUMO5 to substrates *in vitro,* albeit at a reduced rate compared with AtSUMO1/2 (Castano‐Miquel *et al*., 2011). AtSUMO5 is also a poor substrate for the known Arabidopsis ULPs (Chosed *et al*., 2006; Colby *et al*., 2006). As the birth of the *SUMO5/SUL*‐γC gene lineage was close to At‐γ, additional gene copies of the SUMO machinery were probably present in this ancestral species. In line with this, additional *SCE1* gene copies exist in extant eudicot genomes, but not Arabidopsis (Novatchkova *et al*., 2012). By contrast, outside the Plant kingdom, *SCE1* is mostly present as a single gene (Knobbe *et al*., 2015). It will be interesting to examine whether these additional *SCE1* copies have co‐evolved with certain *SUL* genes and have composed novel conjugation pathways.

Remarkably, history has repeated itself in the case of Brassicaceae *SUMO1*/*2,* that is, they exemplify At‐α duplicates that have descended from one of the two archetype eudicot *SUMO* genes. *SUMO1/2* appear to be strictly conserved in Brassicaceae, which implies that they act non‐redundantly and have subfunctionalization in their expression pattern. We have shown previously that this gene pair exhibits tissue‐specific gene expression in Arabidopsis (Van den Burg *et al*., 2010). This agrees with the notion that the loss of *cis*‐regulatory elements allows gene retention as a result of subfunctionalization (Haberer *et al*., 2004), a situation that is reminiscent of the mammalian SUMO2/3 (Wang *et al*., 2014). However, reverse genetics have indicated that, at least in Arabidopsis, *AtSUMO1/2* act redundantly, as the knockout of either gene does not cause growth defects, whereas the double mutant is embryo lethal (Saracco *et al*., 2007). Overexpression of either *SUMO* gene triggers defence activation, whereas expression of dominant‐negative variants activates, even more strongly, plant defence (Van den Burg *et al*., 2010). Combined, *SUMO1/2* appear to represent an example of a gene pair whose expression is dosage balance sensitive (Birchler & Veitia, 2007; De Smet *et al*., 2013). The cause of the dominant‐negative effect is unclear, but, in particular, ULP activity can be inhibited by SUMO overexpression (Mukhopadhyay & Dasso, 2007). By contrast, increased E2 activity via the overexpression or additional SCE1 gene copies appears not to be detrimental to plants (Novatchkova *et al*., 2012; Tomanov *et al*., 2012).

*SUMO3* emerged from a TD of *SUMO2* shortly after At‐α, but before radiation of the Brassicaceae crown group. This TD truncated the *SUMO2* promoter (*c*. 381 bp in Arabidopsis, TAIR), which might be causal to *SUMO2* subfunctionalization. This extra *SUMO* copy rapidly diverged over a short period, yielding *SUMO3*. Genetic studies have indicated that *AtSUMO3* is not essential as the knockout is viable (Van den Burg *et al*., 2010). Moreover, *SUMO3* is frequently deleted, converted back to *SUMO2* or pseudogenized in other Brassicaceae (Fig. 6). Yet, *SUMO3* appears to have neofunctionalized in Arabidopsis, as the gene product has been reported to specifically interact with the salicylic acid receptor NPR1 (Saleh *et al*., 2015) and its expression is transiently induced by this hormone (Van den Burg *et al*., 2010).

Sequence fingerprints were found for both pseudogenes *SUMO4* and *SUMO7* in different Brassicaceae, including *A. arabicum*. In fact, *SUMO4* evolved from a *SUMO‐*γA copy in a recent ancestor of Brassicaceae, whereas *SUMO7* potentially emerged from a segmental duplication involving *SUMO1* in that period (Fig. 8). During this time, the Brassicaceae lineage underwent the At‐α WGD, which might have increased the *SUMO* gene copy number and its protein levels. As a consequence, WGD might have incited neutral selection pressure on *SUMO4* and *SUMO7*, resulting in sequence divergence followed by their pseudogenization. Although the *SUMO‐*γA duplicates were lost/pseudogenized, we noted that the At‐α gene pair that emerged from the *SUMO‐*γB gene subfunctionalized, resulting in *AtSUMO1*/*2*. Both of these observations agree with the notion that housekeeping genes are frequently seen to revert to the singleton state, or subfunctionalize in terms of expression on WGDs (De Smet *et al*., 2013).

Interestingly*,* grasses also contain a diversifying multigene locus that encompasses a tandem array of *SUMO* paralogues (Srilunchang *et al*., 2010). Future studies should help to resolve how this *DSUL/GSUL2* locus emerged. This multigene locus is subject to active TDs and gene rearrangements, resulting in functional head‐to‐tail gene fusions of *SUL* domains. As the number of TDs and UBL repeats in the concatemers vary between closely related species and individual UBL repeats of maize DSUL and rice concatemers group with different clades in the ML tree, it is highly likely that these genomic rearrangements and gene fusions have occurred very recently. This locus exemplifies how di‐ubiquitin‐like proteins ISG15, FAT10 and RUB1 might have evolved in various eukaryotes (Mergner & Schwechheimer, 2014; Basler *et al*., 2015; Radoshevich *et al*., 2015). Related to this, it has been reported that *SCE1* is duplicated in grasses (Novatchkova *et al*., 2012) and that two distinct phylogenetic subclades are retained, suggesting that *GSUL1*,* GSUL2* and/or *DSULs* could potentially have co‐evolved with this divergent *SCE1* orthogroup in monocots.

We have found that, in plants, WGDs followed by TDs are important drivers for *SUMO* paralogue evolution. For example, the pan‐eudicot palaeohexaploidy event has yielded a widespread locus that acts as ‘hotspot’ for *SUMO* paralogue evolution in eudicots, whereas, in Brassicaceae, the paralogue *SUMO3* only emerged after a WGD followed by a TD of one duplicate. Despite these cases of paralogue evolution, we have found that the *SUMO* gene copy number appears to have reverted to a singleton state in plants, and the retained archetype *SUMOs* have subfunctionalized in terms of their expression pattern and not in terms of their sequence.

## Author contributions

H.A.vdB. and M.E.S. designed the research. H.A.vdB. and V.H. carried out data analysis and interpretation. H.A.vdB., V.H. and G.V. performed the research. H.A.vdB., V.H. and M.E.S. wrote the manuscript.

## Supporting information

Please note: Wiley Blackwell are not responsible for the content or functionality of any supporting information supplied by the authors. Any queries (other than missing material) should be directed to the *New Phytologist* Central Office.

**Fig. S1** Frequency distribution of the mean synonymous substitution value per synonymous site (*K*s) for the retained gene duplicates in the AtSUMO1 and AtSUMO2 duplication block.
**Fig. S2** Brassicaceae *SUMO5* evolved from an ancient *Small Ubiquitin‐Like Modifier* (*SUMO*)*‐like* paralog found in eudicots.
**Fig. S3** Brassicaceae *SUMO4* originates from an archetype *Small Ubiquitin‐Like Modifier* (*SUMO*) that diversified after the split of Brassicaceae and Cleomaceae.
**Fig. S4** Maximum likelihood (ML) tree of Brassicaceae *SUMO1*,* SUMO2* and *SUMO3* genes, indicating that *Aethionema arabicum SUMO2* groups sister to the *SUMO2* and *SUMO3* clades.
**Fig. S5** Gene tree of the individual ubiquitin‐like modifier (UBL) domains of the *DiSUMO‐like* (DSUL) locus in grasses with gene identifiers and bootstrap support values indicated.
**Table S1** List of the different plant genomes used in this study
**Table S2** Annotation of the Brassicaceae *Small Ubiquitin‐Like Modifier (SUMO)* paralogues, including expression details.
**Table S3** Gene IDs of the dicot *Small Ubiquitin‐Like Modifier*/*SUMO‐like* (*SUMO/SUL*) genes studied here in the three different genomic regions: *SUMO*‐γA (AtSUMO4), *SUMO*‐γB (AtSUMO1/2) and *SUL*‐γC (AtSUMO5)
**Table S4** Summary of the synteny between *Arabidopsis lyrata SUMO5* (ID: 16062200; Al4g17860) and the Small Ubiquitin‐Like Modifier (SUMO) and SUMO‐like (SUL) genes of the basal eudicots grape vine (*Vitis vinifera*) and eucalyptus (*Eucalyptus grandis*)
