# Supplementary material for: Whole‐genome duplications followed by tandem duplications drive diversification of the protein modifier SUMO in Angiosperms
Source: New Phytol. 2016 Mar 2;211(1):172–85. doi: 10.1111/nph.13911 (PMC6680281; doi:10.1111/nph.13911)
Supplement: Supplementary file 1 — Fig. S1 Frequency distribution of the mean synonymous substitution value per synonymous site (Ks) for the retained gene duplicates in the AtSUMO1 and AtSUMO2 duplication block. Fig. S2 Brassicaceae SUMO5 evolved from an ancient Small Ubiquitin‐Like Modifier (SUMO)‐like paralog found in eudicots. Fig. S3 Brassicaceae SUMO4 originates from an archetype Small Ubiquitin‐Like Modifier (SUMO) that diversified after the split of Brassicaceae and Cleomaceae. Fig. S4 Maximum likelihood (ML) tree of Brassicaceae SUMO1, SUMO2 and SUMO3 genes, indicating that Aethionema arabicum SUMO2 groups sister to the SUMO2 and SUMO3 clades. Fig. S5 Gene tree of the individual ubiquitin‐like modifier (UBL) domains of the DiSUMO‐like (DSUL) locus in grasses with gene identifiers and bootstrap support values indicated. Table S1 List of the different plant genomes used in this study Table S2 Annotation of the Brassicaceae Small Ubiquitin‐Like Modifier (SUMO) paralogues, including expression details. Table S3 Gene IDs of the dicot Small Ubiquitin‐Like Modifier/SUMO‐like (SUMO/SUL) genes studied here in the three different genomic regions: SUMO‐γA (AtSUMO4), SUMO‐γB (AtSUMO1/2) and SUL‐γC (AtSUMO5) Table S4 Summary of the synteny between Arabidopsis lyrata SUMO5 (ID: 16062200; Al4g17860) and the Small Ubiquitin‐Like Modifier (SUMO) and SUMO‐like (SUL) genes of the basal eudicots grape vine (Vitis vinifera) and eucalyptus (Eucalyptus grandis) [file NPH-211-172-s001.pdf]

New Phytologist

***New Phytologist* Supporting Information**

Article title: **Whole-genome duplications followed by tandem-duplications drive diversification of the protein modifier SUMO in Angiosperms**

Authors: Valentin Hammoudi, Georgios Vlachakis, M. Eric Schranz and Harrold A. van den Burg

Article acceptance date: 10 January 2016

The following Supporting Information is available for this article:

Fig. S1 Frequency distribution of the synonymous substitutions per synonymous site (Ks) for the retained ninety gene duplicates in the *AtSUMO1* and *-2* duplication block.

Fig. S2 Brassicaceae *SUMO5* evolved from an ancient *SUMO-like* paralog found in eudicots (see separate Excel file).

Fig. S3 Brassicaceae *SUMO4* originates from an archetype *SUMO* that diversified after the spit of Brassicaceae and Cleomaceae.

Fig. S4 Maximum likelihood (ML) tree of Brassicaceae *SUMO1, -2,* and *-3* genes indicating that *Aethionema arabicum SUMO2* groups sister to the *SUMO2* and *SUMO3* clades.

Fig. S5 Gene tree of the individual UBL domains of DSUL locus in grasses with gene identifiers and bootstrap support values indicated.

Table S1 List of the different plant genomes used in this study.

Table S2 Annotation of the Brassicaceae SUMO paralogs including expression details.

Table S3 Gene IDs of the here studied dicot *SUMO/SUL* genes in the three different genomic regions: *SUMO*-γA (AtSUMO4), *SUMO*-γB (AtSUMO1/2), and *SUL*-γC (AtSUMO5).

Table S4 Summary of the synteny between *A. lyrata SUMO5* (ID: 16062200; Al4g17860) and the SUMO and SUL genes of the basal eudicots grape vine (*V. vinifera*) and eucalyptus (*E. grandis*).

Fig. S1 Frequency distribution of the synonymous substitutions per synonymous site (Ks) for the retained 90 gene duplicates in the *AtSUMO1* (At4g26840) and *-2* (At5g55160) duplication block. The mean Ks of the *AtSUMO1*<->*AtSUMO2* block is calculated using the 90 gene pairs. For comparison, the mean Ks is given for the At-α and At-β WGDs duplication blocks in *A. thaliana* (Kagale *et al*., 2014). The bin sizes depicted is 0.1 number of substitutions per synonymous site with the first bin ranging from 0 to 0.1 substitutions per synonymous site. De data is obtained from the Plant Genome Duplication Database.

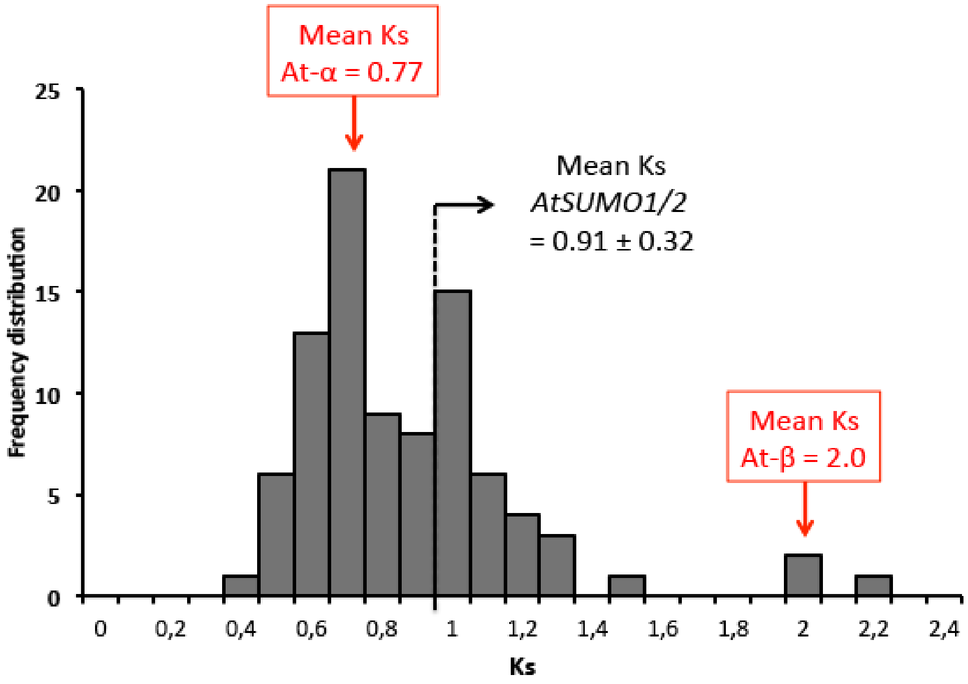

Reference

Kagale S, Robinson SJ, Nixon J, Xiao R, Huebert T, Condie J, Kessler D, Clarke WE, Edger PP, Links MG *et al*. 2014. Polyploid evolution of the Brassicaceae during the Cenozoic era. *Plant Cell* **26**: 2777–2791.

Fig. S2 Brassicaceae *SUMO5* evolved from an ancient *SUMO-like* paralog found in eudicots.
(a) Schematic representation of the gene synteny between the *SUMO5* genomic regions of Arabidopsis (At2g32765), *T. hassleriana* (*Th15853* loci), and eucalyptus (*E. grandis*) *SUL* gene *Eucgr.H00049*. *SUMO5* is syntenic paralog of a divergent *SUL* gene in eucalyptus. Apparently, Arabidopsis *SUMO5* and *Eucgr.H00049* have diversified since their split from their last common ancestor. The genomic position on three different contigs is indicated.
(b) Taxonomic tree with the position of grape, eucalyptus (lineage Myrtales) and the true Rosids (Eurosids), which includes Fabidae (strawberry, soybean) and Malvidae (poplar, Brassicaceae, and *T. hassleriana*).

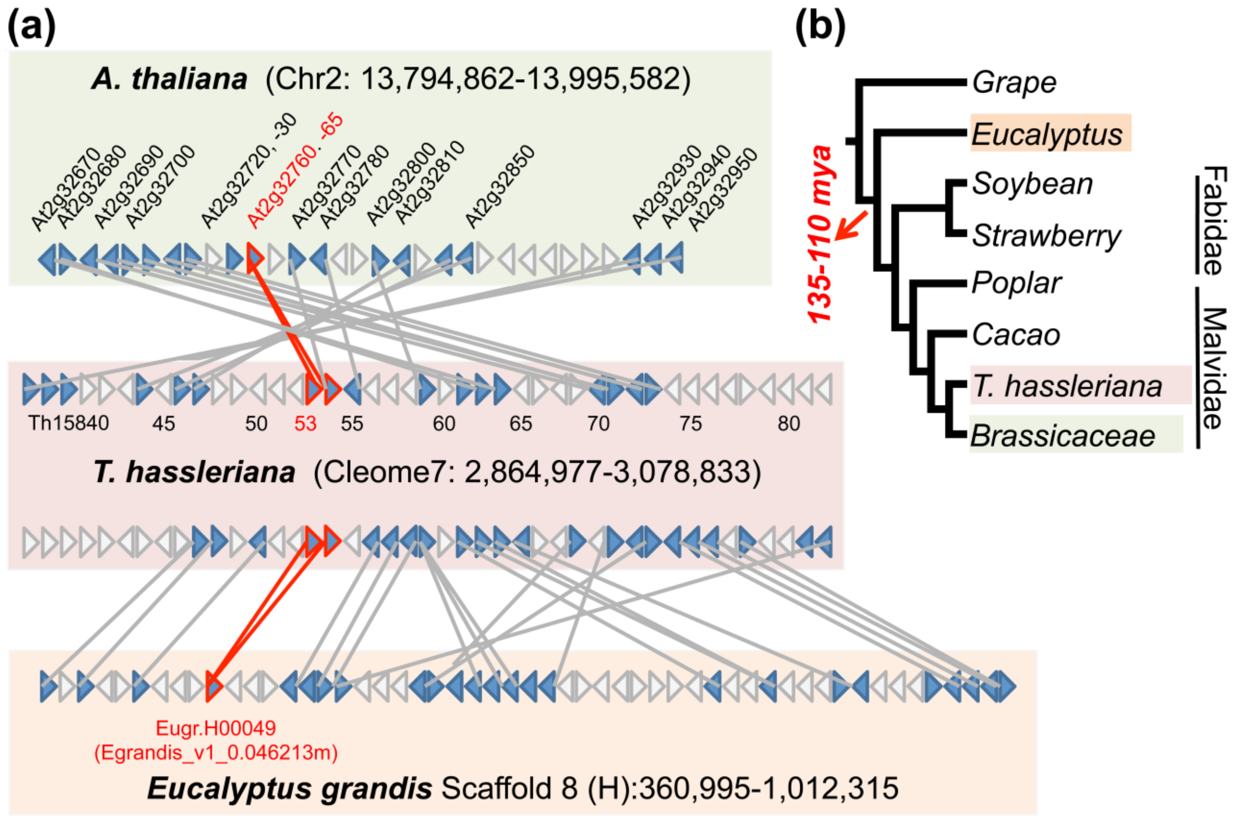

Figure S2

Fig. S3 Brassicaceae *SUMO4* originates from an archetype *SUMO* that diversified after the spit of Brassicaceae and Cleomaceae. Genomic collinearity between the *AtSUMO4* and *-6* (At5g48700, -710) locus in *A. thaliana* and the *Th26484*, *-85*, and *-86* locus in *T. hassleriana* indicates that these five genes represent syntenic paralogues. *Th26486* represents an incomplete tandem duplication of *Th26484* or *-85* genes in the genome assembly, while *Th26484* and *-85* appear to be functional *SUMO* genes.

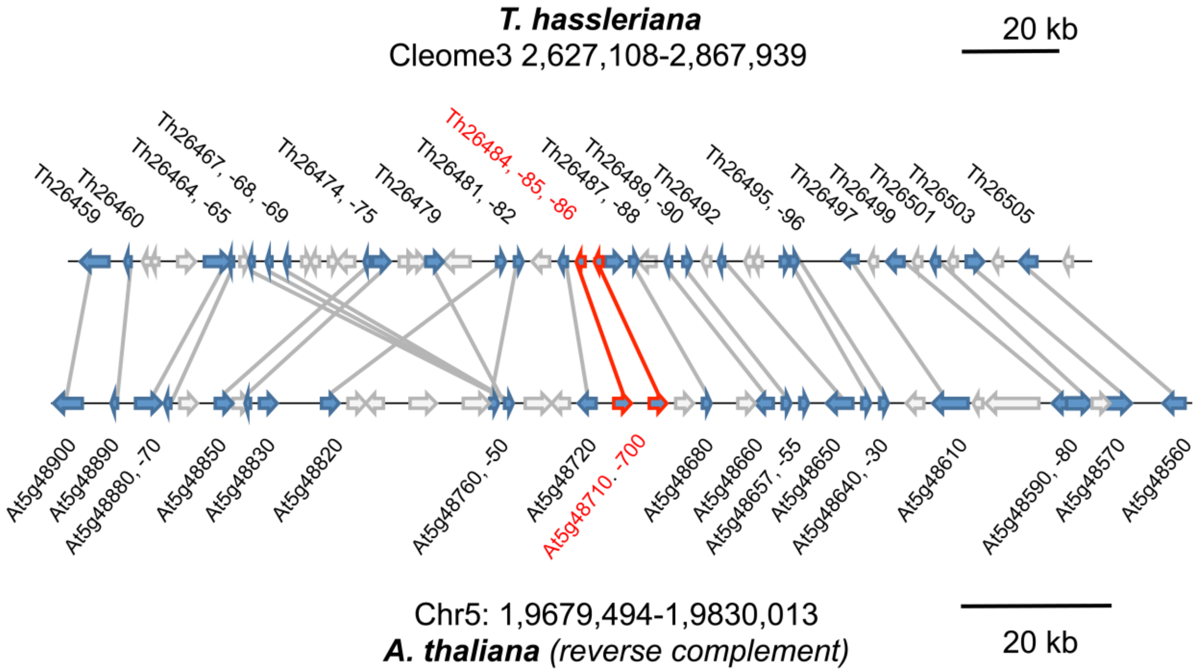

Figure S3

Fig. S4 Maximum likelihood (ML) tree of Brassicaceae *SUMO1, -2,* and *-3* genes indicating that *Aethionema arabicum SUMO2* groups sister to the *SUMO2* and *SUMO3* clades. The tandem duplication of *SUMO2::SUMO3* occurred after the split of Aethionema genus. Both *SUMO1/2* are conserved at the sequence level (short branch lengths) across Brassicaceae, while *SUMO3* has diversified, been lost, or pseudogenized during the same period. A gene conversion event in *E. salsugineum* has converted *SUMO3* in a *-2* paralog (gene ID Thhalv10015081); which is marked with an asterisks (*). Numbers on the nodes represent bootstrap support values (%) of the individual braches. The scale bar corresponds to the number of nucleotide substitutions per site. The *SUMO1/2* sequences from *T. hassleriana* and *Cleome gynandra* were used as outgroup.

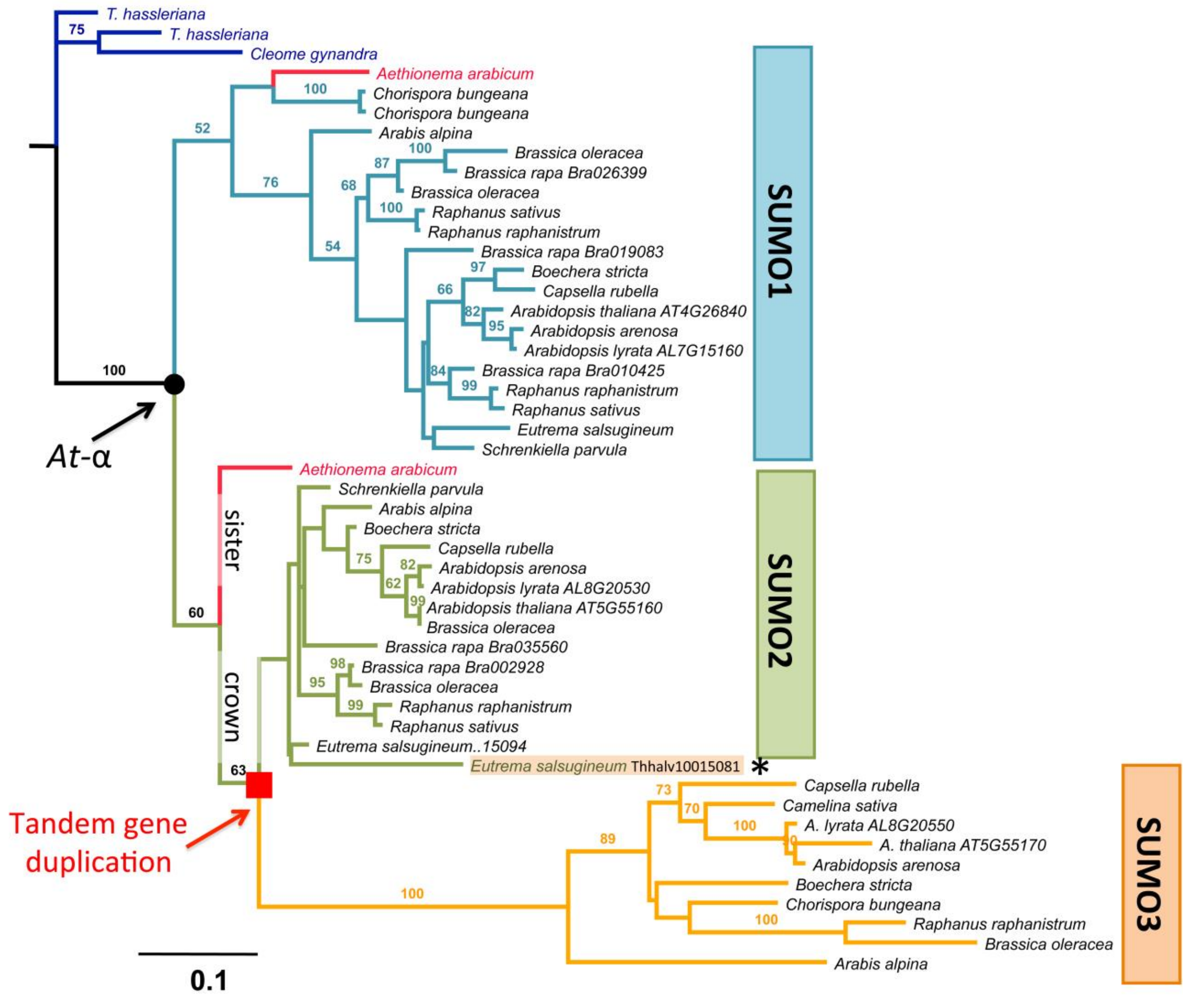

Figure S4

Fig. S5 Gene tree of the individual UBL domains of *DSUL* locus in grasses with gene identifiers and bootstrap support values indicated.

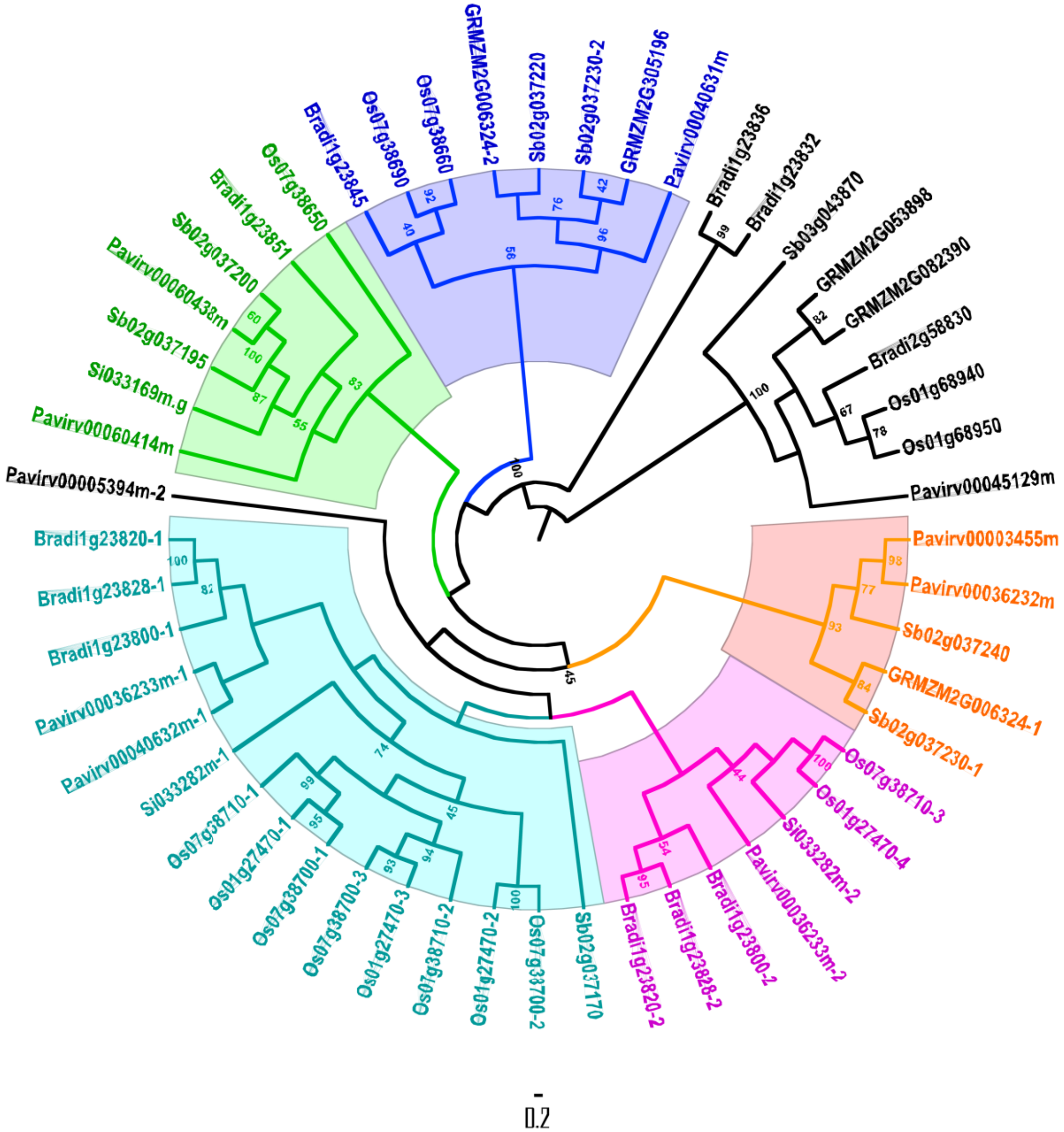

Figure S5

Table S1 List of the different plant genomes used in this study.

Species Name	Common name	Number of Archetype SUMOs	Number of SUMO Paralogs	Genome version	Source	Website	Ref.
**Brassicaceae**							
Aethionema arabicum	N/A	2 (*)	2	v2.5	CoGE	genomevolution.org/	[1]
Arabidopsis arenosa	Sand Rock-cress	2 (*)	4	-	Luca Comai (UC Davis, USA)	comaiwiki.genomecenter.ucdavis.edu	N/A
Arabidopsis halleri	N/A	2	4	v.1.1	Phytozome 10.1	phytozome.jgi.doe.gov	N/A
Arabidopsis lyrata	Lyrata rockcress	3 (*,#)	5	v1.0	PGDD, Phytozome 10.1	chibba.agtec.uga.edu/duplication/, phytozome.jgi.doe.gov	[2]
Arabidopsis thaliana	Arabidopsis	2 (*,#)	6	TAIR10	TAIR	www.arabidopsis.org	[3]
Arabis alpina	Alpine Rock Cress	2 (*)	3	V4	NCBI	NCBI ID: GCA_000733195.1	[4]
Boechera stricta	Drummond's rockcress	2 (*)	4	v1.2	Phytozome 10.1	phytozome.jgi.doe.gov	N/A
Brassica napus	Rapeseed	N/A	N/A	N/A	JCVI	brassica.jcvi.org/cgi-bin/brassica/gbrowse.cgi	N/A
Brassica oleracea	Cauliflower	5 (*,#)	6	v2.1	BRAD	brassicadb.org,	[5]
Brassica rapa	Turnip	5 (*,#)	7	v1.3	BRAD	brassicadb.org,	[6]
Capsella rubella	Capsella	2 (*,#)	4	v1.0	Phytozome 10.1	phytozome.jgi.doe.gov	[7]
Chorispora bungeana	Blue mustard	N/A (*)	N/A	N/A	NCBI WGS, TSA	NCBI ID: GCA_000715565.1	[8]
Eutrema salsugineum (syn. *Thellungielle halophilla*)	N/A	3 (*)	3	v1.0	Phytozome 10.1	phytozome.jgi.doe.gov	[9]
Leavenworthia alabamica	Rollins Alabama gladecress	N/A	N/A	v0.2	CoGE	genomevolution.org/	[1]
Neslia Paniculata	Ballmustard	N/A	N/A	N/A	CoGE	genomevolution.org/	N/A
Raphanus raphanistrum	Wild radish	N/A (*)	N/A	v1.0	RadishBase (EST)	bin/radish/index.cgi	[10]
Raphanus sativus	Radish	N/A (*)	N/A	v1.0	Raphanus sativus genome database, NCBI (TSA)	bioinfo.bti.cornell.edu/cgi-bin, radish.kazusa.or.jp/blast.html, Genbank: JW988067-JW999999, KA000001-KA089547	[11]
Schrenkiella parvula	N/A	2 (*)	2	v2	CoGE,	genomevolution.org/,	[12]

(syn. *Thellungiella parvula*)					Thellungiella.org	Thellungiella.org/	
Sisymbrium irio	London rocket	N/A	N/A	V2.5	CoGE	genomevolution.org/	[1]
**Dicots (non-Brassicaceae)**							
Amborella trichopoda	Amborella	1	0	v1.0	AGD	www.amborella.org	[13]
Beta vulgaris	Sugar beat	1 (#)	5	RefBeet-1.1	PGDD	chibba.agtec.uga.edu/duplication/, bvseq.molgen.mpg.de/index.shtml	[14]
Cajanus cajan	Pigeon pea	1 (#)	ND	Nov 2011	PGDD	chibba.agtec.uga.edu/duplication/	[15]
Carica papaya	Papaya	2 (*,#)	0	ASGPBv0.4	PGDD	chibba.agtec.uga.edu/duplication/,	[16]
Cicer arietinum	Chickpea	3 (#)	1	v1.0	PGDD	chibba.agtec.uga.edu/duplication/, legumeinfo.org/	[17]
Citrullus lanatus	Watermelon	2(#)	1	v1.0	PGDD	chibba.agtec.uga.edu/duplication/	[18]
Citrus sinensis	Sweet orange	2 (*,#)	2	v1.1	PGDD, Phytozome 10.1	chibba.agtec.uga.edu/duplication/, phytozome.jgi.doe.gov	[19]
Cucumis sativus	Cucumber	2 (*,#)	1	v1.0	PGDD, Phytozome 10.1	chibba.agtec.uga.edu/duplication/, phytozome.jgi.doe.gov, www.icugi.org/cgi-bin/IcuGI/index.cgi	[20]
Eucalyptus grandis	Eucalyptus, Flooded gum	3 (#)	4	v1.1	PGDD, CoGE, Phytozome 10.1	chibba.agtec.uga.edu/duplication/, genomevolution.org/	[21]
Fragaria vesca	Strawberry	3 (*,#)	3	v1.1	PGDD, Phytozome 10.1	chibba.agtec.uga.edu/duplication/, phytozome.jgi.doe.gov	[22]
Glycine max	Soybean	3(*,#)	3	Wm82.a2.v1	PGDD, Phytozome 10.1	chibba.agtec.uga.edu/duplication/, www.icugi.org/cgi-bin/IcuGI/index.cgi	[23]
Gossypium raimondii	Cotton	6 (*,#)	4	v2.1	PGDD, CoGE	chibba.agtec.uga.edu/duplication/, genomevolution.org/	[24]
Malus domestica	Apple	6 (*)	N/A	v1.0	PGDD	chibba.agtec.uga.edu/duplication/,	[25]
Medicago truncatula	Barrel medic	2 (#)	6	Mt4.0v1	PGDD	chibba.agtec.uga.edu/duplication/	[26]
Nelumbo nucifera	Sacred lotus	2 (#)	2	v1.0	PGDD	chibba.agtec.uga.edu/duplication/, NCBI: PRJNA16800	[27]
Phaseolus vulgaris	Common bean	2 (*,#)	1	v1.0	PGDD	chibba.agtec.uga.edu/duplication/	[28]
Populus trichocarpa	Western poplar	4 (*,#)	0	v3.0	PGDD	chibba.agtec.uga.edu/duplication/	[29]
Prunus mume	Chinese plum	3 (#)	2	v1.0	PGDD	chibba.agtec.uga.edu/duplication/	[30]
Prunus persica	Peach	3 (*,#)	5	v1.0	PGDD	chibba.agtec.uga.edu/duplication/	[31]
Pyrus bretschneideri	Pear	5 (#)	2	v1.0	PGDD	chibba.agtec.uga.edu/duplication/	[32]
Ricinus communis	Castor bean	2 (*,#)	1	v0.1	PGDD	chibba.agtec.uga.edu/duplication/	[33]

Tarenaya hassleriana (syn. Cleome spinosa)	Prickly Spider Flower	5 (*)	2	v4	CoGE	genomevolution.org/	[34]
Theobroma cacao	Cacao	2 (*,#)	3	v1.1	PGDD, CoGE, Phytozome 10.1	chibba.agtec.uga.edu/duplication/, phytozome.jgi.doe.gov	[35]
Vitis vinifera	Grapevine	3 (*,#)	2	Genoscope (Aug 2007)	PGDD, Phytozome 10.1	chibba.agtec.uga.edu/duplication/, phytozome.jgi.doe.gov	[36]
**Asterids**							
Actinidia chinensis	Kiwifruit	3 (#)	1	May 2013	PGDD	chibba.agtec.uga.edu/duplication/	[37]
Capsicum annuum	Hot pepper	2 (#)	2	v1.55	PGDD	chibba.agtec.uga.edu/duplication/ peppersequence.genomic.cn/	[38]
Mimulus guttatus	Monkey flower	2 (*)	4	V2.0	Phytozome 10.1	phytozome.jgi.doe.gov	[39]
Solanum lycopersicum	Tomato	3 (*,#)	2	v2.4	PGDD, SOL Genomics Network	chibba.agtec.uga.edu/duplication/, solgenomics.net/	[40]
Solanum tuberosum	Potato	2 (*,#)	5	v3.4	PGDD	chibba.agtec.uga.edu/duplication/	[41]
**Monocots**							
Brachypodium distachyon	Purple false brome	1 (*)	8	v2.1	PGDD	chibba.agtec.uga.edu/duplication/	[42]
Oryza sativa	Rice	2 (*)	6	v7.0	PGDD, Phytozome 10.1	chibba.agtec.uga.edu/duplication/, phytozome.jgi.doe.gov	[43]
Setaria italica	Foxtail millet	ND	ND	v2.1	Phytozome 10.1	phytozome.jgi.doe.gov	[44]
Sorghum bicolor	Sorghum	1 (*)	8	v2.1	PGDD	chibba.agtec.uga.edu/duplication/	[45]
Musa acuminata	Banana	4 (*)	ND	July 2012	PGDD	chibba.agtec.uga.edu/duplication/	[46]
Panicum virgatum	Switchgrass	N/A	N/A	v1.1	Phytozome 10.1	phytozome.jgi.doe.gov	N/A
Zea mays	Maize	2 (*)	4	v6a	PGDD	chibba.agtec.uga.edu/duplication/	[47]
Elaeis guineensis	Oil palm	3	ND	v2.0	PGDD	chibba.agtec.uga.edu/duplication/	[48]

Footnotes:

N/A, (1) Not or poorly annotated genome assembly limited our estimate of the number of SUMOs/SULs. (2) Publication unknown.
ND, Not determined.
*, Archetype SUMO sequences from this species were used to generate the gene tree in Figure 2.
#, SUMO & SUL sequences from this species are represented in Figure 5, where they form jointly the SUMO-SUL network.

References

1. **Haudry, A., A.E. Platts, E. Vello, D.R. Hoen, M. Leclercq, R.J. Williamson, E. Forczek, Z. Joly-Lopez, J.G. Steffen, K.M. Hazzouri *et al*. 2013**. An atlas of over 90,000 conserved noncoding sequences provides insight into crucifer regulatory regions. *Nature Genetics* **45**: 891–898.
2. **Hu, T.T., P. Pattyn, E.G. Bakker, J. Cao, J.F. Cheng, R.M. Clark, N. Fahlgren, J.A. Fawcett, J. Grimwood, H. Gundlach *et al*. 2011**. The *Arabidopsis lyrata* genome sequence and the basis of rapid genome size change. *Nature Genetics* **43**: 476–481.
3. **The Arabidopsis Genome Initiative. 2000**. Analysis of the genome sequence of the flowering plant *Arabidopsis thaliana*. *Nature* **408**: 796–815.
4. **Willing, E.-M., V. Rawat, T. Mandáková, F. Maumus, G.V. James, K.J.V. Nordström, C. Becker, N. Warthmann, C. Chica, B. Szarzynska *et al*. 2015**. Genome expansion of *Arabis alpina* linked with retrotransposition and reduced symmetric DNA methylation. *Nature Plants* **1**: 14023.
5. **Parkin, I.A., C. Koh, H. Tang, S.J. Robinson, S. Kagale, W.E. Clarke, C.D. Town, J. Nixon, V. Krishnakumar, S.L. Bidwell *et al*. 2014**. Transcriptome and methylome profiling reveals relics of genome dominance in the mesopolyploid *Brassica oleracea*. *Genome Biology* **15**: R77.
6. **Wang, X., H. Wang, J. Wang, R. Sun, J. Wu, S. Liu, Y. Bai, J.H. Mun, I. Bancroft, F. Cheng *et al*. 2011**. The genome of the mesopolyploid crop species *Brassica rapa*. *Nature Genetics* **43**: 1035–1039.
7. **Slotte, T., K.M. Hazzouri, J.A. Agren, D. Koenig, F. Maumus, Y.L. Guo, K. Steige, A.E. Platts, J.S. Escobar, L.K. Newman *et al*. 2013**. The *Capsella rubella* genome and the genomic consequences of rapid mating system evolution. *Nature Genetics* **45**: 831–835.
8. **Zhao, Z., L. Tan, C. Dang, H. Zhang, Q. Wu, and L. An 2012**. Deep-sequencing transcriptome analysis of chilling tolerance mechanisms of a subnival alpine plant, *Chorispora bungeana*. *BMC Plant Biology* **12**: 222.
9. **Yang, R., D.E. Jarvis, H. Chen, M.A. Beilstein, J. Grimwood, J. Jenkins, S. Shu, S. Prochnik, M. Xin, C. Ma *et al*. 2013**. The reference genome of the halophytic plant *Eutrema salsugineum*. *Frontiers in Plant Science* **4**: 46.
10. **Moghe, G.D., D.E. Hufnagel, H. Tang, Y. Xiao, I. Dworkin, C.D. Town, J.K. Conner, and S.H. Shiu 2014**. Consequences of whole-genome triplication as revealed by comparative genomic analyses of the wild radish *Raphanus raphanistrum* and three other Brassicaceae species. *Plant Cell* **26**: 1925–1937.
11. **Kitashiba, H., F. Li, H. Hirakawa, T. Kawanabe, Z. Zou, Y. Hasegawa, K. Tonosaki, S. Shirasawa, A. Fukushima, S. Yokoi *et al*. 2014**. Draft sequences of the radish (*Raphanus sativus* L.) genome. *DNA Research* **21**: 481–490.
12. **Oh, D.H., H. Hong, S.Y. Lee, D.J. Yun, H.J. Bohnert, and M. Dassanayake 2014**. Genome structures and transcriptomes signify niche adaptation for the multiple-ion-tolerant extremophyte *Schrenkiella parvula*. *Plant Physiology* **164**: 2123–2138.
13. **Amborella Genome Project. 2013**.The Amborella genome and the evolution of flowering plants. *Science* **342**: 1241089.

14. **Dohm, J.C., A.E. Minoche, D. Holtgrawe, S. Capella-Gutierrez, F. Zakrzewski, H. Tafer, O. Rupp, T.R. Sorensen, R. Stracke, R. Reinhardt *et al*. 2014**. The genome of the recently domesticated crop plant sugar beet (*Beta vulgaris*). *Nature* **505**: 546–549.
15. **Varshney, R.K., W. Chen, Y. Li, A.K. Bharti, R.K. Saxena, J.A. Schlueter, M.T. Donoghue, S. Azam, G. Fan, A.M. Whaley *et al*. 2012**. Draft genome sequence of pigeonpea (*Cajanus cajan*), an orphan legume crop of resource-poor farmers. *Nature Biotechnology* **30**: 83–89.
16. **Ming, R., S. Hou, Y. Feng, Q. Yu, A. Dionne-Laporte, J.H. Saw, P. Senin, W. Wang, B.V. Ly, K.L. Lewis *et al*. 2008**. The draft genome of the transgenic tropical fruit tree papaya (*Carica papaya* Linnaeus). *Nature* **452**: 991–996.
17. **Varshney, R.K., C. Song, R.K. Saxena, S. Azam, S. Yu, A.G. Sharpe, S. Cannon, J. Baek, B.D. Rosen, B. Tar'an *et al*. 2013**. Draft genome sequence of chickpea (*Cicer arietinum*) provides a resource for trait improvement. *Nature Biotechnology* **31**: 240–246.
18. **Guo, S., J. Zhang, H. Sun, J. Salse, W.J. Lucas, H. Zhang, Y. Zheng, L. Mao, Y. Ren, Z. Wang *et al*. 2013**. The draft genome of watermelon (*Citrullus lanatus*) and resequencing of 20 diverse accessions. *Nature Genetics* **45**: 51–58.
19. **Xu, Q., L.L. Chen, X. Ruan, D. Chen, A. Zhu, C. Chen, D. Bertrand, W.B. Jiao, B.H. Hao, M.P. Lyon *et al*. 2013**. The draft genome of sweet orange (*Citrus sinensis*). *Nature Genetics* **45**: 59–66.
20. **Huang, S., R. Li, Z. Zhang, L. Li, X. Gu, W. Fan, W.J. Lucas, X. Wang, B. Xie, P. Ni *et al*. 2009**. The genome of the cucumber, *Cucumis sativus* L. *Nature Genetics* **41**: 1275–1281.
21. **Myburg, A.A., D. Grattapaglia, G.A. Tuskan, U. Hellsten, R.D. Hayes, J. Grimwood, J. Jenkins, E. Lindquist, H. Tice, D. Bauer *et al*. 2014**. The genome of *Eucalyptus grandis*. *Nature* **510**: 356–362.
22. **Shulaev, V., D.J. Sargent, R.N. Crowhurst, T.C. Mockler, O. Folkerts, A.L. Delcher, P. Jaiswal, K. Mockaitis, A. Liston, S.P. Mane *et al*. 2011**. The genome of woodland strawberry (*Fragaria vesca*). *Nature Genetics* **43**: 109–116.
23. **Schmutz, J., S.B. Cannon, J. Schlueter, J. Ma, T. Mitros, W. Nelson, D.L. Hyten, Q. Song, J.J. Thelen, J. Cheng *et al*. 2010**. Genome sequence of the palaeopolyploid soybean. *Nature* **463**: 178–183.
24. **Paterson, A.H., J.F. Wendel, H. Gundlach, H. Guo, J. Jenkins, D. Jin, D. Llewellyn, K.C. Showmaker, S. Shu, J. Udall *et al*. 2012**. Repeated polyploidization of *Gossypium* genomes and the evolution of spinnable cotton fibres. *Nature* **492**: 423–427.
25. **Velasco, R., A. Zharkikh, J. Affourtit, A. Dhingra, A. Cestaro, A. Kalyanaraman, P. Fontana, S.K. Bhatnagar, M. Troggio, D. Pruss *et al*. 2010**. The genome of the domesticated apple (*Malus* × *domestica* Borkh.). *Nature Genetics* **42**: 833–839.
26. **Young, N.D., F. Debelle, G.E. Oldroyd, R. Geurts, S.B. Cannon, M.K. Udvardi, V.A. Benedito, K.F. Mayer, J. Gouzy, H. Schoof *et al*. 2011**. The *Medicago* genome provides insight into the evolution of rhizobial symbioses. *Nature* **480**: 520–524.
27. **Ming, R., R. VanBuren, Y. Liu, M. Yang, Y. Han, L.T. Li, Q. Zhang, M.J. Kim, M.C. Schatz, M. Campbell *et al*. 2013**. Genome of the long-living sacred lotus (*Nelumbo nucifera* Gaertn.). *Genome Biology* **14**: R41.

28. **Schmutz, J., P.E. McClean, S. Mamidi, G.A. Wu, S.B. Cannon, J. Grimwood, J. Jenkins, S. Shu, Q. Song, C. Chavarro *et al*. 2014**. A reference genome for common bean and genome-wide analysis of dual domestications. *Nature Genetics* **46**: 707–713.
29. **Tuskan, G.A., S. Difazio, S. Jansson, J. Bohlmann, I. Grigoriev, U. Hellsten, N. Putnam, S. Ralph, S. Rombauts, A. Salamov *et al*. 2006**. The genome of black cottonwood, *Populus trichocarpa* (Torr. & Gray). *Science* **313**: 1596–1604.
30. **Zhang, Q., W. Chen, L. Sun, F. Zhao, B. Huang, W. Yang, Y. Tao, J. Wang, Z. Yuan, G. Fan *et al*. 2012**. The genome of *Prunus mume*. *Nature Communications* **3**: 1318.
31. **Verde, I., A.G. Abbott, S. Scalabrin, S. Jung, S. Shu, F. Marroni, T. Zhebentyayeva, M.T. Dettori, J. Grimwood, F. Cattonaro *et al*. 2013**. The high-quality draft genome of peach (*Prunus persica*) identifies unique patterns of genetic diversity, domestication and genome evolution. *Nature Genetics* **45**: 487–494.
32. **Wu, J., Z. Wang, Z. Shi, S. Zhang, R. Ming, S. Zhu, M.A. Khan, S. Tao, S.S. Korban, H. Wang *et al*. 2013**. The genome of the pear (*Pyrus bretschneideri* Rehd.). *Genome Research* **23**: 396–408.
33. **Chan, A.P., J. Crabtree, Q. Zhao, H. Lorenzi, J. Orvis, D. Puiu, A. Melake-Berhan, K.M. Jones, J. Redman, G. Chen *et al*. 2010**. Draft genome sequence of the oilseed species *Ricinus communis*. *Nature Biotechnology* **28**: 951–956.
34. **Cheng, S., E. van den Bergh, P. Zeng, X. Zhong, J. Xu, X. Liu, J. Hofberger, S. de Bruijn, A.S. Bhide, C. Kuelahoglu *et al*. 2013**. The *Tarenaya hassleriana* genome provides insight into reproductive trait and genome evolution of crucifers. *Plant Cell* **25**: 2813–2830.
35. **Argout, X., J. Salse, J.M. Aury, M.J. Guiltinan, G. Droc, J. Gouzy, M. Allegre, C. Chaparro, T. Legavre, S.N. Maximova *et al*. 2011**. The genome of *Theobroma cacao*. *Nature Genetics* **43**: 101–108.
36. **Jaillon, O., J.M. Aury, B. Noel, A. Policriti, C. Clepet, A. Casagrande, N. Choisne, S. Aubourg, N. Vitulo, C. Jubin *et al*. 2007**. The grapevine genome sequence suggests ancestral hexaploidization in major angiosperm phyla. *Nature* **449**: 463–467.
37. **Huang, S., J. Ding, D. Deng, W. Tang, H. Sun, D. Liu, L. Zhang, X. Niu, X. Zhang, M. Meng *et al*. 2013**. Draft genome of the kiwifruit *Actinidia chinensis*. *Nature Communications* **4**: 2640.
38. **Kim, S., M. Park, S.I. Yeom, Y.M. Kim, J.M. Lee, H.A. Lee, E. Seo, J. Choi, K. Cheong, K.T. Kim *et al*. 2014**. Genome sequence of the hot pepper provides insights into the evolution of pungency in *Capsicum* species. *Nature Genetics* **46**: 270–278.
39. **Hellsten, U., K.M. Wright, J. Jenkins, S. Shu, Y. Yuan, S.R. Wessler, J. Schmutz, J.H. Willis, and D.S. Rokhsar 2013**. Fine-scale variation in meiotic recombination in *Mimulus* inferred from population shotgun sequencing. *Proceedings of the National Academy of Sciences, USA* **110**: 19478–19482.
40. **The Tomato Genome Consortium. 2012**. The tomato genome sequence provides insights into fleshy fruit evolution. *Nature* **485**: 635–641.
41. **Potato Genome Sequencing Consortium. 2011**. Genome sequence and analysis of the tuber crop potato. *Nature* **475**: 189–195.

42. **The International Brachypodium Initiative. 2010**. Genome sequencing and analysis of the model grass *Brachypodium distachyon*. *Nature* **463**: 763–768.
43. **International Rice Genome Sequencing Project. 2005**. The map-based sequence of the rice genome. *Nature* **436**: 793–800.
44. **Bennetzen, J.L., J. Schmutz, H. Wang, R. Percifield, J. Hawkins, A.C. Pontaroli, M. Estep, L. Feng, J.N. Vaughn, J. Grimwood *et al*. 2012**. Reference genome sequence of the model plant Setaria. *Nature Biotechnology* **30**: 555–561.
45. **Paterson, A.H., J.E. Bowers, R. Bruggmann, I. Dubchak, J. Grimwood, H. Gundlach, G. Haberer, U. Hellsten, T. Mitros, A. Poliakov *et al*. 2009**. The *Sorghum bicolor* genome and the diversification of grasses. *Nature*, **457**: 551–556.
46. **D'Hont, A., F. Denoeud, J.M. Aury, F.C. Baurens, F. Carreel, O. Garsmeur, B. Noel, S. Bocs, G. Droc, M. Rouard *et al*. 2012**. The banana (*Musa acuminata*) genome and the evolution of monocotyledonous plants. *Nature* **488**: 213–217.
47. **Schnable, P.S., D. Ware, R.S. Fulton, J.C. Stein, F. Wei, S. Pasternak, C. Liang, J. Zhang, L. Fulton, T.A. Graves *et al*. 2009**. The B73 maize genome: complexity, diversity, and dynamics. *Science* **326**: 1112–1115.
48. **Singh, R., M. Ong-Abdullah, E.T. Low, M.A. Manaf, R. Rosli, R. Nookiah, L.C. Ooi, S.E. Ooi, K.L. Chan, M.A. Halim *et al*. 2013**. Oil palm genome sequence reveals divergence of interfertile species in Old and New worlds. *Nature* **500**: 335–339.

New Phytologist

Table S2 Annotation of the Brassicaceae SUMO paralogs including expression details. (See the separate Excel file.)

Table S3 Gene IDs of the here studied dicot *SUMO/SUL* genes in the three different genomic regions: SUMO-γA (AtSUMO4), SUMO-γB (AtSUMO1/2), and *SUL*-γC (AtSUMO5). Three ancient SUMO gene clusters are found in the genomes of eudicots. One cluster represents an ancient highly divergent SUMO-like UBL family (*AtSUMO5*), while the other two genomic clusters contain archetype *SUMO* genes (*AtSUMO1*) except for Brassicaceae (*AtSUMO4*). The gene IDs given are obtained from CoGE (*genomevolution.org/*), PGDD (*chibba.agtec.uga.edu/duplication/*), or Phytozome 10 (*phytozome.jgi.doe.gov/*) data repositories. SUL sequences are indicated by a red gene ID.

Species	Taxons				Syntenic homologs		
					**SUMOγ–B**	**SUMOγ–A**	***SULγ–C***
Nelumbo nucifera	**Proteales**				*ND*[1]	NNU_022372-RA	
Beta vulgaris	**Caryophyllales**				Bv4_087850_dyxu	*ND*	Bv3_068150_orpx #
Actinidia chinensis					*ND*	Achn127311<br>Achn307081[2]	Achn266161::<br>Achn266171
Capsicum annuum					CA07g19790	*ND*	CA03g01160 #
Solanum lycopersici	**Asterids**				Solyc07g049360.2<br>Solyc07g064880.2<br>Solyc12g006010.1[3]	Solyc09g059970.2	Solyc09g091890.2 #
Solanum tuberosum					PGSC0003DMC400038444<br>PGSC0003DMC400001311<br>PGSC0003DMC400024516[3]	*ND*	PGSC0003DMC400054530<br>::<br>PGSC0003DMC400054531<br>#
Vitis vinifera	**Vitales**				GSVIVT01030502001	GSVIVT01003301001[4]<br>GSVIVG01003307001[4]	GSVIVT01021058001 #
Eucalyptus grandis	**Myrtales**				Eucgr.K00755::<br>Eucgr.K00756<br>(Egrandis_v1_0.037737m::<br>Egrandis_v1_0.033394m)	Eucgr.H00789<br>(Egrandis_v1_0.039699m)	**Eucgr.H00049** #<br>(Egrandis_v1_0.046213m)
Phaseolus vulgaris					Phvul.006G047600	Phvul.008G129000	Phvul.003G291000 #
Glycine max	**Fabales**				Glyma.08G320500	Glyma.08G350600<br>Glyma.18G165200	Glyma.05G154000 #,<br>Glyma.08G111700::<br>Glyma.08G111800 #
Fragaria vesca					mrna01213,<br>mrna03443	mrna17600	mrna06159
Pyrus bretschneideri					Pbr005170.1<br>Pbr024463.1<br>Pbr039768.1	Pbr012819.1<br>Pbr025468.1[2]<br>Pbr033049.1	Pbr030915.1::<br>Pbr030916.1 #
Prunus mume	**Rosales**	**Fabidae**			Pm015148,<br>Pm011903<br>Pm012555	Pm020340	Pm009507[2]
Prunus persica				**Eudicots**	ppa012976m,<br>ppa023777m::<br>ppa013753m::ppa013880m<br>ppa023122m	ppa013826m	ppa014098m
Citrullus lanatus	**Cucurbitales**				Cla005336<br>Cla013517<br>Cla016281	*ND*	*ND*
Cucumis sativus					16952982::16952983,<br>16967643,<br>16982462::16982463	*ND*	*ND*
Ricinus communis	**Malpighiales**		**Eurosids**		29762.m000499	30204.m001744	**29904.m002927 #**
Populus trichocarpa					Potri.002G224700::<br>Potri.002G224800,<br>Potri.014G158300	Potri.014G190300	*ND*
Gossypium raimondii					Gorai.001G095200[5]<br>Gorai.001G216800[5]<br>Gorai.009G442500<br>Gorai.013G158500	Gorai.004G058800	**Gorai.009G349100[4] #**
Theobroma cacao	**Brassicales**	**Malvidae**			Thecc1EG002167<br>(Tc01_g017640)	Thecc1EG002904<br>(Tc01_g021470)	**Thecc1EG020307 #,<br>Thecc1EG020389::<br>Thecc1EG020390 #**<br>(Tc04_g021070::<br>Tc04_g021080::<br>Tc04_g021100)
Citrus sinensis<br>*Citrus clementina*					orange1.1g034926m<br>Ciclev10017283m	orange1.1g034043m<br>Ciclev10022993m	**orange1.1g048514m::<br>orange1.1g045424m[3] #,<br>Ciclev10006329m[3]**
Carica papaya					evm.TU.supercontig_132.24	evm.TU.supercontig_8.130	*ND*
Tarenaya hassleriana					Th15536<br>Th11421<br>Th20205[6]	Th20051,<br>Th26484::Th26485::<br>Th26486 (partial SUM4	**Th15853[7]**

						like)	
Brassicaceae					**SUMO1** (At4g26840), **SUMO2::SUMO3** (At5g55160:At5g55170)	**SUMO4::SUMO6** (AT5G48700, -710)	**SUMO5** (At2g32765)

1, *ND* indicates that a syntenic paralog was not found in the full genome assembly.
2, Gene fusion or misannotation in the genome assembly.
3, SUMO-like sequences, Solanaceae-specific gene duplication.
4, Not contained in PGDD block.
5, This genomic region appears to correspond to SUMO5 in Arabidopsis, but it resembles the SUMO1 genomic region in Papaya.
6, Potential pseudogene – sequence is incomplete and diglycine motif is absent.
7, Missanotation in the genome assembly, i.e. gene fusion of a *SUMO5* paralog with an upstream gene.
#, Indicates that the diglycine motif is present.
::, Indicates a tandem duplication of SUMO(-like) genes at one locus.

Table S4 Summary of the synteny between *A. lyrata SUMO5* (ID: 16062200; Al4g17860) and the SUMO and SUL genes of the basal eudicots grape vine (*V. vinifera*) and eucalyptus (*E. grandis*).

Species	Gene ID		Number of Anchors	
	SUMO1 SUMO-γB	***SUMO5 SUL-γC***	***SUMO1 SUMO-γB***	***SUMO5 SUL-γC***
V. vinifera	GSVIVT01030502001	GSVIVT01021058001	14	30
E. grandis	Eucgr.K00755	Eucgr.H00049	7	12
